# *Aspergillus nidulans* protein kinase A plays an important role in cellulase production

**DOI:** 10.1186/s13068-015-0401-1

**Published:** 2015-12-18

**Authors:** Leandro José de Assis, Laure Nicolas Annick Ries, Marcela Savoldi, Thaila Fernanda dos Reis, Neil Andrew Brown, Gustavo Henrique Goldman

**Affiliations:** Departamento de Ciências Farmacêuticas, Faculdade de Ciências Farmacêuticas de Ribeirão Preto, Universidade de São Paulo, Av. do Café S/N, CEP 14040-903, Ribeirão Preto, São Paulo, Brazil; Plant Biology and Crop Science, Rothamsted Research, Harpenden, Herts AL5 2JQ UK

**Keywords:** *Aspergillus nidulans*, Protein kinase A, Carbon catabolite repression, Glucose metabolism, Cellulose

## Abstract

**Background:**

The production of bioethanol from lignocellulosic feedstocks is dependent on lignocellulosic biomass degradation by hydrolytic enzymes. The main component of lignocellulose is cellulose and different types of organisms are able to secrete cellulases. The filamentous fungus *Aspergillus nidulans* serves as a model organism to study cellulase production and the available tools allow exploring more in depth the mechanisms governing cellulase production and carbon catabolite repression.

**Results:**

In *A. nidulans*, microarray data identified the cAMP-dependent protein kinase A (PkaA) as being involved in the transcriptional modulation and the production of lignocellulolytic enzymes in the presence of cellulose. Deletion of *pkaA* resulted in increased hydrolytic enzyme secretion, but reduced growth in the presence of lignocellulosic components and various other carbon sources. Furthermore, genes involved in fungal development were increased in the Δ*pkaA* strain, probably leading to the increased hyphal branching as was observed in this strain. This would allow the secretion of higher amounts of proteins. In addition, the expression of SynA, encoding a V-SNARE synaptobrevin protein involved in secretion, was increased in the *ΔpkaA* mutant. Deletion of *pkaA* also resulted in the reduced nuclear localization of the carbon catabolite repressor CreA in the presence of glucose and in partial de-repression when grown on cellulose. PkaA is involved in the glucose signaling pathway as the absence of this protein resulted in reduced glucose uptake and lower hexokinase/glucokinase activity, directing the cell to starvation conditions. Genome-wide transcriptomics showed that the expression of genes encoding proteins involved in fatty acid metabolism, mitochondrial function and in the use of cell storages was increased.

**Conclusions:**

This study shows that PkaA is involved in hydrolytic enzyme production in *A. nidulans*. It appears that this protein kinase blocks the glucose pathway, hence forcing the cell to change to starvation conditions, increasing hydrolytic enzyme secretion and inducing the usage of cellular storages. This work uncovered new regulatory avenues governing the tight interplay between the metabolic states of the cell, which are important for the production of hydrolytic enzymes targeting lignocellulosic biomass. Deletion of *pkaA* resulted in a strain with increased hydrolytic enzyme secretion and reduced biomass formation.

**Electronic supplementary material:**

The online version of this article (doi:10.1186/s13068-015-0401-1) contains supplementary material, which is available to authorized users.

## Background

Lignocellulosic plant biomass represents a cheap, abundant and renewable carbon feedstock for next-generation biofuels and green technologies. In nature, microbes such as bacteria and fungi are able to deconstruct and grow on plant cell wall polysaccharides [1, 2]. The enzymes responsible for the degradation, or modification, of these plant polysaccharides, are broadly termed carbohydrate-active enzymes (CAZymes) [3–5]. Industrial cocktails of microbial CAZymes are used to release fermentable sugars from lignocellulose for bioethanol production. However, inefficiencies in microbial enzyme production and the conversion of all the types of sugars found in lignocellulose into bioethanol prevent the widespread application of such technologies.

The ascomycete *Aspergillus nidulans* is a model filamentous fungus commonly used to study the regulation and secretion of lignocellulolytic enzymes [6]. During growth on lignocellulose, the fungus secretes an array of different enzymes, which act in synergy to degrade the recalcitrant substrate. In the presence of glucose, the carbon source favored by most organisms, the secretion of these plant cell wall-degrading enzymes and the utilization of alternative carbon sources are repressed by carbon catabolite repression (CCR), which is mediated by the CreA transcriptional repressor [7]. In the presence of glucose, CreA has been shown to repress the transcription of genes encoding enzymes important for the utilization of alternative carbon sources [8], such as proline, ethanol, xylan [9], cellulose [10, 11] and arabinan [12, 13].

The reversible phosphorylation of target proteins is performed by the opposing activities of kinases and phosphatases. This post-translational mechanism is important for modulating protein structure, function and location, playing a crucial role in many cell signaling mechanisms including the regulation of CCR [14]. In *Saccharomyces cerevisiae* the AMP-activated protein kinase Snf1p regulates carbon assimilation, the usage of alternative carbon sources and glucose de-repression [15]. In *S. cerevisiae*, Mig1-mediated CCR is controlled by Snf1p. In the presence of low levels of glucose, Snf1p phosphorylates and releases the DNA bound Mig1p, which is subsequently exported from the nucleus, alleviating the repression of glucose-repressed genes [16]. Deletion of *SNF1* homologues in filamentous fungi, including *A. nidulans*, has also been shown to influence CreA de-repression and reduce hydrolytic enzyme production [8, 17–19].

The cAMP-dependent protein kinase A (PKA) is another important player involved in coordinating primary metabolism, CCR and fungal growth. In *A. nidulans*, the two catalytic subunits of PKA are encoded by *pkaA* and *pkaB*, with PkaA performing the major role within the cell. PkaA positively controls germination and vegetative growth-related functions in response to various nutrients via the G protein-coupled receptor (GPCR) and Ras signaling pathways [20–22]. Upon activation of the GPCR or Ras pathways adenylate cyclase increases cAMP production, which in turn binds to the regulatory subunit (PkaR) of PkaA, releasing the active catalytic subunit to phosphorylate downstream targets [21, 23]. In conidia, cAMP-dependent PKA activation results in isotropic growth, germ tube formation and trehalose degradation [23–25]; in *S. cerevisiae* PKA activity is activated in response to glucose and promotes glycolysis and fermentation and in *A. fumigatus* PKA activity was increased in the presence of glucose compared to glycerol [26]. Deletion of the genes *pkaC1/pkaC2* in *A. fumigatus* renders the fungus unable to grow on glucose, further supporting a role for PKA in glucose metabolism [27]. The addition of glucose to the growth media, increased cAMP levels which in turn activated PKA in yeast [28], *A. nidulans* and *A. fumigatus* [23, 29, 30]. However, PKA activity can still be detected in the absence of the adenylate cyclase, indicating the existence of a cAMP-independent route for PKA activation [8]. In *Trichoderma reesei,* adenylate cyclase and protein kinase A were shown to be involved in the regulation of cellulase gene expression as deletion of both adenylate cyclase and PKA resulted in increased levels of cellulase gene expression [31].

This work carried out a detailed characterization of the involvement of PkaA in carbon source utilization. This study demonstrates that PkaA is involved in regulating CreA cellular localization and glucose signaling. PkaA expression was modulated in the absence of any carbon source and/or in the presence of recalcitrant carbon sources like cellulose, showing a transient expression. Furthermore, deletion of *pkaA* reduced glucose uptake and phosphorylation by hexo/glucokinases activities. In the absence of this protein kinase, the energetic status of the cell is directed towards carbon starvation resulting in increased hydrolytic enzyme production.

## Results

### Deletion of *pkaA* resulted in early increased expression of genes encoding hydrolytic enzymes and carbon metabolism-specific transcription factors

Microarray analyses were used to investigate the genome-wide effect of the deletion of *pkaA* during growth on complete media (a repressing condition) and crystalline cellulose, avicel (a de-repressing condition). Strain-specific transcriptional differences were identified. Although the growth of Δ*pkaA* mutant was dramatically reduced in liquid glucose-containing minimal media (data not shown), the growth rate was comparable to the wild-type strain when grown in liquid complete YG media (24 h, wild type = 0.116 ± 0.010 g/10^7^ conidia; Δ*pkaA* = 0.167 ± 0.1018 g/10^7^ conidia). Thus, the wild-type and Δ*pkaA* strains were grown for 24 h in complete media and then transferred to minimal media supplemented with 1 % (w/v) avicel for 8 h and 24 h. Genes that were differentially expressed between post-transfers to avicel, in an individual strain, were identified (*p* < 0.01). Genes which were up- or down-regulated in the Δ*pkaA* and wild-type strains were submitted for CAZy (Carbohydrate-Active enZYmes) [32] and MIPS FunCat categorization [33].

**Fig. 1:**
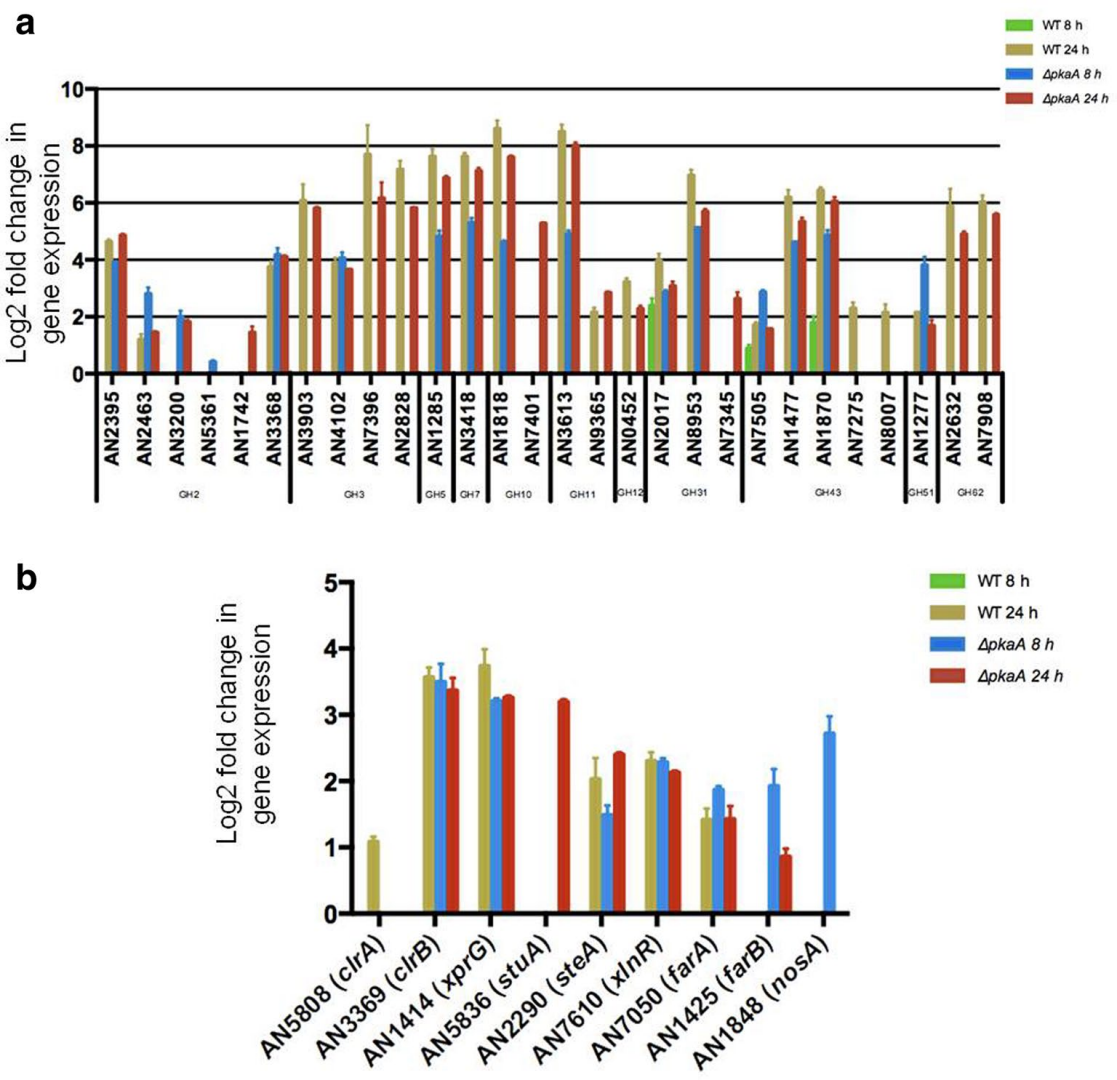
**a** Deletion of *pkaA* results in an earlier onset of glycoside hydrolase (GH) gene expression. Gene expression values are shown for various enzymes belonging to different GH families in the wild-type (WT) and Δ*pkaA* strains when grown in biological triplicates in cellulose-rich media for 8 and 24 h. **b** Genes encoding transcription factors important for cellulose, hemicellulose and fatty acid utilization as well as for mediating the carbon starvation response are up-regulated at an earlier time point in the Δ*pkaA* strain. Experiments were carried out in biological triplicates and all changes in the levels of gene expression listed here have a statistical significance of *p* < 0.01

The microarray data were submitted for analysis of log2 fold change in the expression of CAZyme (Carbohydrate-Active enZyme)-encoding genes. CAZymes are enzymes which modify, break down or synthesize carbohydrate structures and consist of the glycoside hydrolases (GHs), carbohydrate esterases (CEs), polysaccharide lyases (PLs), auxiliary activities (AA) and glycosyltransferases (GTs) (http://www.cazy.org). In this dataset, GH-encoding genes presented 62 % (Additional file 1: Table S1) of all the CAZyme-encoding genes whereas the remaining 38 % contained the CEs, PLs, GTs and AAs. GH-encoding genes were classified into their respective families for the wild-type and Δ*pkaA* strains in the above-described conditions. The results indicate that genes encoding GHs are induced much earlier in the Δ*pkaA* strain in the presence of avicel than when compared to the wild-type strain (Fig. 1a). For example, the two endoglucanase-encoding genes *eglA* (GH5) and *eglB* (GH7), which play a major role in cellulose degradation, as well as a putative β-glucosidase-encoding gene (AN4102), were up-regulated ~4.6-, ~4.9- and ~4.1-fold, respectively, after 8 h incubation in cellulose in the Δ*pkaA* strain but not in the wild-type strain (Additional file 1: Table S1). The expression of some GHs, like AN3200 (β-glucuronidase), AN5361 (β-glucuronidase), AN1742 (β-1,4-mannosidase), AN7401 (endo-1,4-β-xylanase) and AN7345 (glucosidase) were modulated only in the Δ*pkaA* strain (8 and 24 h cellulose), the wild-type strain showed specific modulation for AN7275 (1,4-β-xylosidase) and AN8007 (endo-1,5-α-L-arabinosidase).

The above-described results suggest that deletion of *pkaA* has a significant effect on carbon metabolism. Subsequently, to further investigate how PkaA is involved in these cellular processes, the expression of transcription factors involved in carbon metabolism was analyzed (Fig. 1b). The expression of the positive regulators of cellulase and xylanase genes, *clrB* (AN3369) and *xlnR* (AN7610) which are under the control of CCR [34–36], was increased after 8 h growth on avicel in the Δ*pkaA* strain but not in the wild-type strain. However, after 24-h growth on avicel the expression of both transcription factors was similar in both strains.

The transcriptional activators, *farA* and *farB,* regulate genes important for fatty acid utilization [37]. Similar to *clrB* and *xlnR*, the expression of *farB* was increased after 8 h on avicel in the Δ*pkaA* strain only, while *farA* was induced in the Δ*pkaA* strain after 8 h and 24 h but not in the wild-type strain, indicating the activation of fatty acid utilization in the Δ*pkaA* strain. Fatty acids can serve as sole carbon source for fungi and are degraded to acetyl-CoA and other Krebs cycle components, mainly in peroxisomes [37]. Fatty acids have been shown to be important for fungal development and secondary metabolite production. *farA* is the orthologue of *PEX6* in *S. cerevisiae*, a protein required for the import of proteins into the peroxisome [37]. FarB was shown to be important for short-chain fatty acid utilization [37]. Additionally, *xprG* (AN1414), a transcription factor involved in the regulation of extracellular proteases and in the regulation of the carbon starvation response [38], was also up-regulated at an earlier time point during growth on avicel in the Δ*pkaA* strain. The earlier up-regulation of these transcription factors as well as of the genes encoding hydrolytic enzymes suggest that *ΔpkaA* is experiencing increased metabolic stress due to defects in carbon uptake, storage, and sensing, and this is probably impacting enzyme regulation and CCR.

Furthermore, genes which were up- or down-regulated in both strains when compared to control condition (complete medium) were submitted for MIPS FunCat categorization. *ΔpkaA*-specific transcriptional alterations included genes encoding proteins involved in fatty acid metabolism, protein degradation, peroxisomal transport, stress response and biogenesis of vacuoles or lysosomes after 8 h treatment with avicel (Table 1; Additional file 2: Table S2). Categories of genes, which were specifically down-regulated in the Δ*pkaA* strain included proteins involved in the pyruvate dehydrogenase complex (PDC), DNA synthesis and replication, translational control and biogenesis of the nuclear membrane (Table 2; Additional file 2: Table S2). After 24 h growth in avicel, expression of genes belonging to *ΔpkaA*-specific categories remained up-regulated (protein degradation, stress response and biogenesis of vacuole or lysosome) and down-regulated (PDC, protein synthesis, nuclear transport and biogenesis) (Additional file 3: Table S3). In the wild-type strain, genes encoding proteins of the PDC, kinase activator and anion and ion transport were up-regulated and genes encoding proteins involved in electron transport, transcription and vesicular transport and differentiation were down-regulated after 8 h and 24 h in the presence of avicel (Additional file 2: Table S2; Additional file 3: Table S3).

**Table 1:** MIPS functional catalog category classification of all the genes specifically up-regulated in *pkaA* deletion strain

Functional category	Genes	*p* value
01 Metabolism		
01.01.03.01.01 biosynthesis of glutamine	3	0.0382
01.01.09.04 metabolism of phenylalanine	17	0.0260
01.01.09.04.01 biosynthesis of phenylalanine	11	0.0340
01.01.11.02.02 degradation of isoleucine	5	0.0171
01.01.11.03.02 degradation of valine	5	0.0232
01.01.11.04 metabolism of leucine	11	0.0058
01.01.11.04.02 degradation of leucine	9	0.0033
01.02 nitrogen, sulfur and selenium metabolism	43	0.0389
01.05.03 polysaccharide metabolism	41	0.0114
01.05.06 C-2 compound and organic acid metabolism	9	0.0368
01.06.05 fatty acid metabolism	39	0.0035
01.20.01.05 metabolism of sugar alcohols	4	0.0047
01.20.01.09 metabolism of aminoglycoside antibiotics	3	0.0238
01.20.07 metabolism of propionic acid derivatives	2	0.0373
02 Energy		
02.01 glycolysis and gluconeogenesis	18	0.0419
02.07 pentose-phosphate pathway	12	0.0219
02.16 fermentation	28	0.0413
02.16.01 alcohol fermentation	10	0.0230
02.16.03 lactate fermentation	6	0.0069
02.25 oxidation of fatty acids	22	0.0145
14 Protein fate (folding, modification, destination)		
14.07.11.01 autoproteolytic processing	7	0.0128
14.13 protein/peptide degradation	64	0.0002
14.13.01 cytoplasmic and nuclear protein degradation	40	0.0124
14.13.04 lysosomal and vacuolar protein degradation	14	0.0073
14.13.04.02 vacuolar protein degradation	9	0.0097
18 Regulation metabolism and protein function		
18.02.10 regulation of channel activity	2	0.03735
20 Cellular transp., transp. facilities and routes		
20.01.03 C-compound and carbohydrate transport	44	0.03095
20.09.18 cellular import	73	0.01043
20.09.18.07 non-vesicular cellular import	44	0.00332
20.09.03 peroxisomal transport	10	0.00743
30 Cellular communication/signal transduct. mechanism		
30.01.05.03 protease mediated signal transduction	2	0.03735
30.01.09.03 Ca^2+^-mediated signal transduction	8	0.00950
32 Cell rescue, defense and virulence		
32.01.04 pH stress response	3	0.03822
32.01.05 heat-shock response	11	0.00852
32.07.03 detoxification by modification	11	0.03405
32.07.07 oxygen and radical detoxification	11	0.03405
32.07.07.03 glutathione conjugation reaction	4	0.04179
32.07.07.07 superoxide metabolism	4	0.03061
34 Interaction with the environment		
34.01.01.01 homeostasis of metal ions (Na, K, Ca, etc.)	29	0.03479
38 Transposable elements, viral, plasmid protein		
38.07 proteins necessary for transposon movement	4	0.01412
42 Biogenesis of cellular components		
42.19 peroxisome	14	0.00119
42.25 vacuole or lysosome	11	0.02263

**Table 2:** MIPS functional catalog category classification of all the genes specifically down-regulated in the *pkaA* deletion strain

Functional category	Genes	*p* value
01 Metabolism		
01.01 amino acid metabolism	107	0.00013
01.01.03 assim. ammonia, metab. glutamate group	27	0.00201
01.01.03.05 metabolism of arginine	10	0.01657
01.01.03.05.01 biosynthesis of arginine	8	0.00840
01.01.05 metab. urea cycle, creatine and polyamines	9	0.03639
01.01.06.01 metabolism of aspartate	7	0.00523
01.01.06.01.02 degradation of aspartate	5	0.01900
01.01.06.04 metabolism of threonine	7	0.00359
01.01.06.04.01 biosynthesis of threonine	3	0.03204
01.01.06.05 metabolism of methionine	14	0.00040
01.01.06.05.01 biosynthesis of methionine	7	0.02838
01.01.06.05.01.01 biosynthesis of homocysteine	3	0.05076
01.01.09 metabolism of the cysteine - aromatic group	43	0.02539
01.01.11.01 metabolism of alanine	3	0.00784
01.01.11.02 metabolism of isoleucine	10	0.00434
01.01.11.02.01 biosynthesis of isoleucine	8	0.00331
01.01.11.02.02 degradation of isoleucine	5	0.02647
01.01.11.03 metabolism of valine	8	0.02220
01.01.11.03.01 biosynthesis of valine	7	0.00523
01.01.11.03.02 degradation of valine	5	0.03558
01.03.01 purin nucleot/nucleoside/nucleobase metab.	33	0.00029
01.03.07 deoxyribonucleotide metabolism	7	0.01761
01.03.16 polynucleotide degradation	21	0.03254
01.03.16.01 RNA degradation	14	0.02504
01.05.13 transfer of activated C-1 groups	23	0.00011
01.05.13.03 tetrahydrofolate-dependent C-1-transfer	7	0.00237
01.06.06 isoprenoid metabolism	31	0.02022
01.06.06.11 tetracyclic and pentacyclic triterpenes (cholesterin, steroids and hopanoids) metabolism	28	0.00044
01.07 metab. vitamins, cofactors, and prosthetic groups	53	0.00514
01.07.01 biosyn. vitam, cofactors, prosthetic groups	33	0.00305
01.20.19 metabolism of secondary products derived from glycine, l-serine and l-alanine	12	0.00100
01.20.19.01 metabolism of porphyrins	10	0.00134
02 Energy		
02.07.01 pentose-phosphate pathway oxidative branch	2	0.04599
02.08 pyruvate dehydrogenase complex	3	0.05076
02.10 (citrate cycle, Krebs cycle, TCA cycle)	13	0.01823
02.13 respiration	51	0.00123
02.13.01 anaerobic respiration	6	0.00061
02.13.03 aerobic respiration	37	0.00715
10 Cell cycle and DNA processing		
10.01.03 DNA synthesis and replication	31	0.00080
10.01.03.03 ori recognition, priming complex formation	8	0.00630
11 Transcription		
11.02.01 rRNA synthesis	31	0.00153
11.04.01 rRNA processing	89	0.00002
11.04.03 mRNA processing (splicing, 5^-, 3^-end)	63	0.00398
11.04.03.01 splicing	56	0.00235
11.06.01 rRNA modification	12	0.00448
12 Protein synthesis		
12.01 ribosome biogenesis	174	0.00004
12.01.01 ribosomal proteins	126	0.00006
12.04 translation	115	0.00080
12.04.02 translation elongation	9	0.00396
12.04.03 translation termination	3	0.03204
12.07 translational control	23	0.00122
12.10 aminoacyl-tRNA-synthetases	18	0.00100
14 Protein fate (folding, modification, destination)		
14.01 protein folding and stabilization	30	0.00077
14.04 protein targeting, sorting and translocation	59	0.00044
14.10 assembly of protein complexes	56	0.00275
16 Protein binding function or cofactor required (structural or catalytic)		
16.03.01 DNA binding	68	0.05063
16.07 structural protein binding	14	0.02191
16.19.05 GTP binding	26	0.00253
16.21 complex cofactor/cosubstrate/vitamin binding	78	0.00581
16.21.05 FAD/FMN binding	27	0.02943
16.21.17 pyridoxal phosphate binding	16	0.00077
20 Cel. transp., transp. facilities and transp. routes		
20.01.01.01.01 heavy metal ion transport (Cu + , Fe3 +)	15	0.03629
20.01.03.03 C4-transport (malate, succinate, fumarate)	6	0.00356
20.01.21 RNA transport	16	0.01353
20.09.01 nuclear transport	21	0.00100
20.09.04 mitochondrial transport	48	0.00572
20.09.16.05 Type V protein secretion system	2	0.01677
42 Biogenesis of cellular components		
42.10.05 nuclear membrane	8	0.00231
42.10.07 nucleolus	4	0.00028
42.16 mitochondrion	69	0.00004

In summary, deletion of *pkaA* resulted in the up-regulation of degradation pathways and a shift in metabolism to the use of alternative energy sources (e.g., fatty acids), the down-regulation of DNA replication, protein synthesis and the PDC. Furthermore, this microarray data show that deletion of *pkaA* results in increased expression of genes encoding lignocellulosic enzymes, especially after 8 h incubation in cellulose, whereas this response was already reduced after 24 h incubation in cellulose. This indicates a change in the flux of carbon metabolism within the cell.

### PkaA is involved in carbon catabolite repression (CCR)

To validate the microarray data, the wild-type, Δ*pkaA*, Δ*snfA* and the Δ*pkaA* Δ*snfA* (constructed by genetically crossing Δ*pkaA* and Δ*snfA*) strains were grown in complete media for 24 h and then transferred to minimal media supplemented with 1 % avicel or 2 % glucose plus 1 % avicel for 5 days. The growth phenotype of the double mutant was similar to the growth profile of the Δ*pkaA* strain (Additional file 4: Figure S1). The Δ*snfA* strain was included because in *S. cerevisiae,* the antagonism between PKA and Snf1 regulates carbon utilization [39, 40], while in *A. nidulans* SnfA has a great influence on cellulase/xylanase induction and CCR [8, 18, 19].

**Fig. 2:**
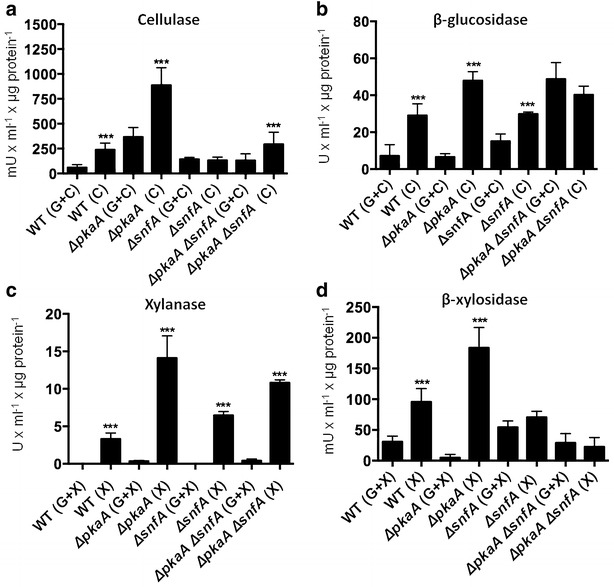
Deletion of pkaA results in an increase in secreted hydrolytic enzymes. **a** Cellulase activities, **b** β-glucosidase activities, **c** xylanase activities and **d** β-xylosidase activities in different strains. Mycelia were grown in complete media for 24 h before being transferred to minimal medium supplemented with 1 % Avicel (C, cellulose) or xylan (X) or to minimal medium supplemented with 2 % glucose and 1 % cellulose or xylan (G + C; G + X) for 5 or 3 days, respectively. Enzymatic activities were determined in culture supernatants. All enzyme activities were normalized by intracellular protein concentration. Experiments were carried out in biological triplicates and the statistical significance of (***) *p* < 0.001 between repressing (G + C; G + X) and de-repressing (C; X) conditions

As expected, the wild-type strain showed increased (more than threefold) cellulase (EGL and CBH) production after 5 days growth on 1 % avicel. Beta-glucosidase (BGL) activity was also measured. Similarly, BGL activity was higher in the Δ*pkaA* mutant than in the wild-type strain (Fig. 2b). This is in agreement with the microarray data, where during early incubation in avicel (e.g., 8 h), the expression of *eglA*, *eglB* and a BGL-encoding gene was ~fourfold higher in the Δ*pkaA* strain than when compared to the wild-type strain. After 24 h though, the expression of these genes was similar between the Δ*pkaA* and wild-type strains.

To confirm whether carbon catabolite repression was active in the absence of *pkaA*, the *pkaA* deletion and wild-type strains were grown in the simultaneous presence of glucose and avicel. Cellulase secretion was repressed in the wild-type strain, whereas the Δ*pkaA* mutant showed a fourfold increase in cellulase production when compared to the wild-type strain in these conditions (Fig. 2a). The Δ*snfA* mutant was used as a negative control and showed only a basal level of cellulase production but no clear induction as was observed for the wild-type strain after transfer to avicel for 5 days. BGL activity induction was observed in the Δ*snfA* strain; however, the enzyme activity was at similar levels than in the wild-type strain. The double Δ*pkaA* Δ*snfA* deletion mutant behaved like the wild-type strain, secreting a similar amount of cellulases in the presence of avicel as the sole carbon source. An increase in the activity of BGL was observed in the double mutant under repressing and de-repressing conditions, showing levels of enzyme activity similar to the *ΔpkaA* mutant under de-repressing conditions. These results suggest that the Δ*pkaA* mutation can suppress the *ΔsnfA* mutation and that both genes are somehow genetically interacting.

To check if de-repression also occurs in the presence of xylan, the Δ*pkaA*, Δ*snfA,* Δ*pkaA* Δ*snfA* and wild-type strains were grown in complete media and subsequently transferred to media containing only 1 % xylan or 1 % xylan supplemented with 2 % glucose for 3 days before xylanase and β-xylosidase (BXL) activities were measured in the culture supernatants. Xylanase activity was increased in the presence of xylan and repressed in the simultaneous presence of xylan and glucose in the wild-type strain. In the Δ*pkaA* mutant, xylanase activity was three times higher than in the wild-type strain in the presence of xylan. This is in agreement with the microarray data where the major xylanase-encoding genes *xlnA* and *xlnC* were up-regulated ~4.6- and ~4.9-fold, respectively, after 8-h incubation in cellulose in the Δ*pkaA* strain but not in the wild-type strain (Additional file 2: Table S2). In contrast, after 24-h incubation in cellulose, *xlnA* and *xlnC* gene expression was similar between the wild-type and Δ*pkaA* strains.

Xylanase activity was also increased in the simultaneous presence of glucose and xylan in the Δ*pkaA* strain than when compared to the wild-type strain (Fig. 2c). Again, this confirms that deletion of *pkaA* increases hydrolytic enzyme secretion. BXL activity was measured in the same conditions and results were similar to the ones shown for BGL activity. BXL activity was higher in the *ΔpkaA* strain than when compared to the wild-type strain in the presence of xylan but not in the simultaneous presence of glucose and xylan (Fig. 2d). There is no difference in BXL activity in the *ΔsnfA* strain and the double mutant in repressing and de-repressing conditions (Fig. 2d).

These enzymatic data validate the microarray and implies that the deletion of *pkaA* renders the fungus partially blind to the presence of glucose, suggesting a role for PkaA in CCR. Additionally, these results also suggest a complex genetic interaction between PkaA and SnfA during cellulase induction and glucose de-repression.

### Deletion of *pkaA* results in reduced CreA nuclear localization upon growth on glucose

The above-mentioned results suggest that PkaA is involved in glucose metabolism and/or CCR. Cellular localization of CreA::GFP in the wild-type and Δ*pkaA* strains was assessed when the strains were grown in minimal media supplemented with 1 % glucose or 1 % avicel. In the wild-type and Δ*pkaA* strains, 96 and 25 % of CreA::GFP localized to the nucleus in the presence of glucose whereas 2 and 20 % of CreA::GFP localized to the nucleus in the presence of avicel (Table 3). These results further indicate that PkaA is involved in CCR as CreA is (partially) unable to localize to the nucleus during repressing conditions in the absence of *pkaA*.

**Table 3:** Percentage of CreA::GFP nuclear localization in different strains under different conditions

Strain	Grown in	Nuclear CreA (%)
*creA::GFP*	1 % Glucose	96
*creA::GFP ΔpkaA*	1 % Glucose	25
*creA::GFP*	1 % Avicel	2
*creA::GFP ΔpkaA*	1 % Avicel	20

Strain	Transfer to	Nuclear CreA (%)
*creA::GFP*	1 % Avicel	17
*creA::GFP ΔpkaA*	1 % Avicel	24
*creA::GFP*	1 % Glucose	100
*creA::GFP ΔpkaA*	1 % Glucose	67

Spores were inoculated in 1 % glucose or avicel for 16 h at 22 °C before being transferred to minimal media supplemented with 1 % avicel for 5 h or before 1 % glucose was added to the overnight avicel cultures for 30 min

### PkaA is involved in protein secretion and hyphal branching

**Fig. 3:**
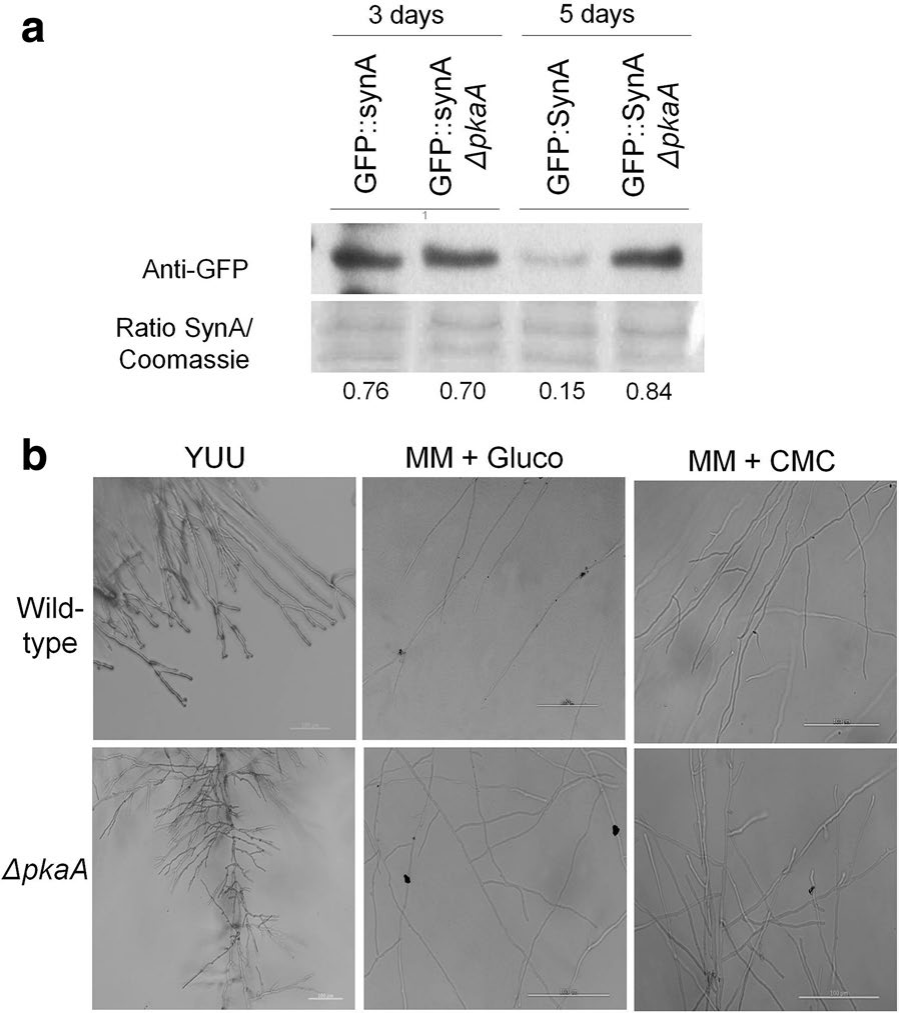
Deletion of *pkaA* results in increased hyphal branching. **a** Western blot of GFP::SynA. Mycelia were grown from spores in complete media for 24 h and then transferred to minimal media supplemented with 1 % cellulose for 3 and 5 days before proteins were extracted. For normalization, a gel was run with the total protein extract and subsequently stained with *Coomassie blue*. **b** Mycelia were grown from spores in complete media (YUU), minimal media supplemented with glucose (MM + Gluco) or minimal media supplemented with CMC carboxymethylcellulose (MM + CMC) for 3 days at 37 °C. Pictures were taken at a ×20 magnification (*scale bar* 100 μm)

As shown by the microarray analysis and enzymatic assays, expression of genes encoding cellulases and xylanases was up-regulated and the secretion of cellulases and xylanases was increased in the Δ*pkaA* strain (Fig. 2). To know whether secretion is specifically responsible for this increase in lignocellulolytic enzyme production or whether it is due to morphological changes, the microarray data were analyzed for the expression of genes involved in protein secretion. Six genes which encode proteins involved in the secretion process were up-regulated after 8-h incubation in cellulose in the Δ*pkaA* strain but not in the wild-type strain. These genes encoded a protein with SNAP receptor activity (AN8488), proteins with transmembrane activity and membrane localization (AN8983, AN5763, AN5559 and AN4019) and a putative transmembrane transporter (AN7295). After 24-h growth in cellulose, the expression of these genes was similar between the Δ*pkaA* and wild-type strains. To further investigate the influence of *pkaA* on secretion, a *ΔpkaA* GFP::SynA strain was generated by crossing the respective parental strains and the amount and fluorescence of GFP::SynA was assessed during growth in cellulose-minimal media. SynA is a V-SNARE synaptobrevin protein involved in the secretion pathway that localizes to the plasma membrane in actively growing hyphal apex [41, 42]. In agreement with the microarray results, Western blot analysis showed that GFP::SynA levels were about fivefold higher in the Δ*pkaA* strain than when compared to the wild-type strain after 5 days of growth in cellulose (Fig. 3a). No differences were observed in GFP::SynA distribution to the hyphal apex of germlings of the parental GFP::SynA and the *ΔpkaA* GFP::SynA strains after growing for 16 h in cellulose (Additional file 4: Figure S2).

Subsequently, the morphology and number of hyphal tips were assessed and evaluated in the Δ*pkaA* strain, as increased protein secretion could correlate with fungal colony morphology. Deletion of *pkaA* resulted in increased branching (and hence an increased number of tips) when grown in complete media and minimal media supplemented with glucose or CMC (Fig. 3b). It appears that the deletion of *pkaA* results in an increased area of secretion which may contribute to the higher amounts of proteins secreted (Additional file 4: Figure S3). At the same time, components of the secretion pathway seem to be up-regulated at early and late time points in the Δ*pkaA* strain, which may also contribute to the observed increase in enzyme secretion. Taken together our results suggest that *A. nidulans* PkaA is important for hyphal branching and secretion.

### PkaA translation is triggered under carbon starvation and carbon catabolite de-repressing conditions

**Fig. 4:**
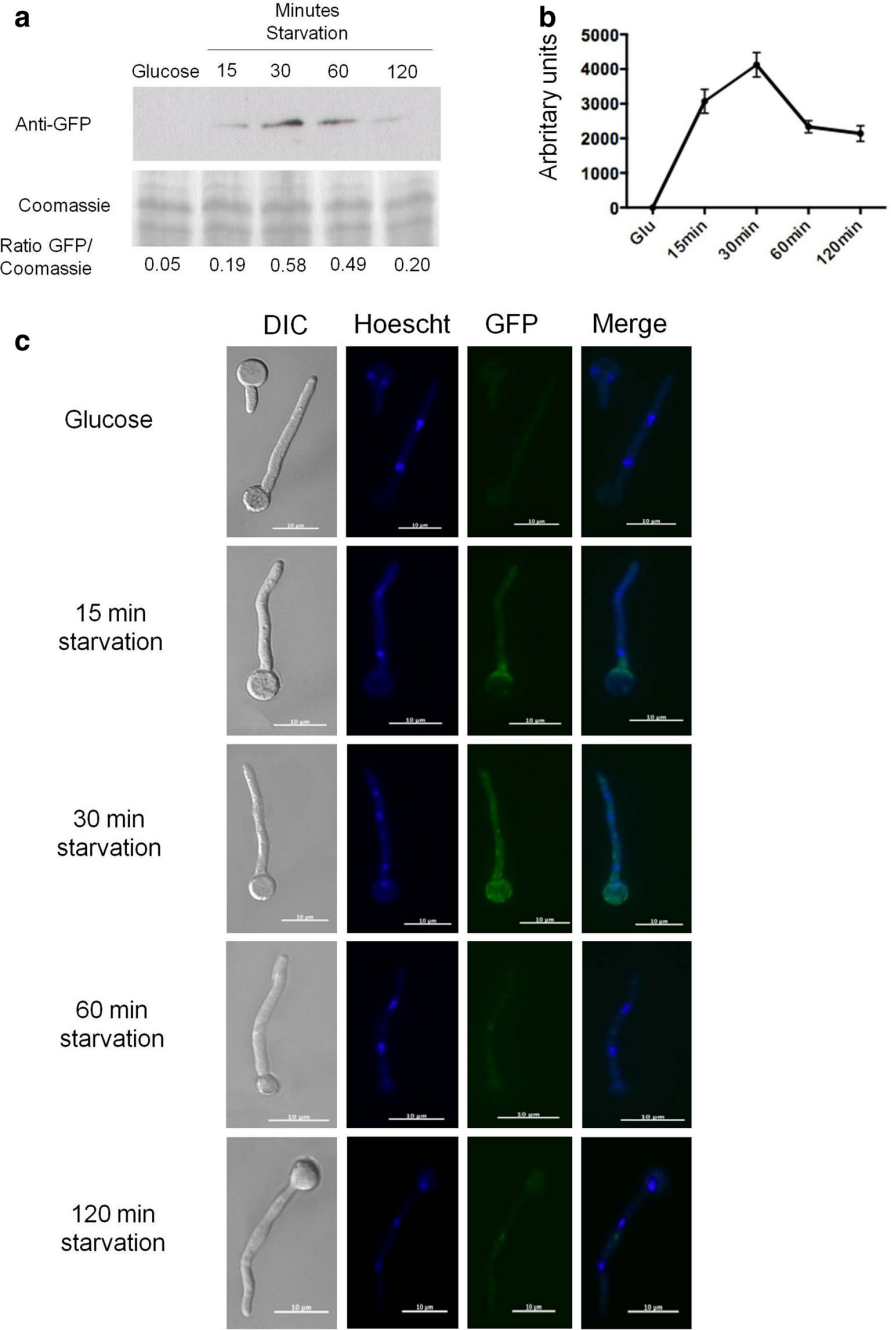
PkaA is involved in the response to carbon starvation. **a** Western blot, **b** PkaA activity and **c** fluorescence microscopy of pkaA::GFP. Mycelia were grown from spores in minimal media supplemented with 1 % glucose for 16 h at 22 °C, washed 2× with water before being transferred to minimal media without any carbon source (starvation) for 15, 30, 60 and 120 min

**Fig. 5:**
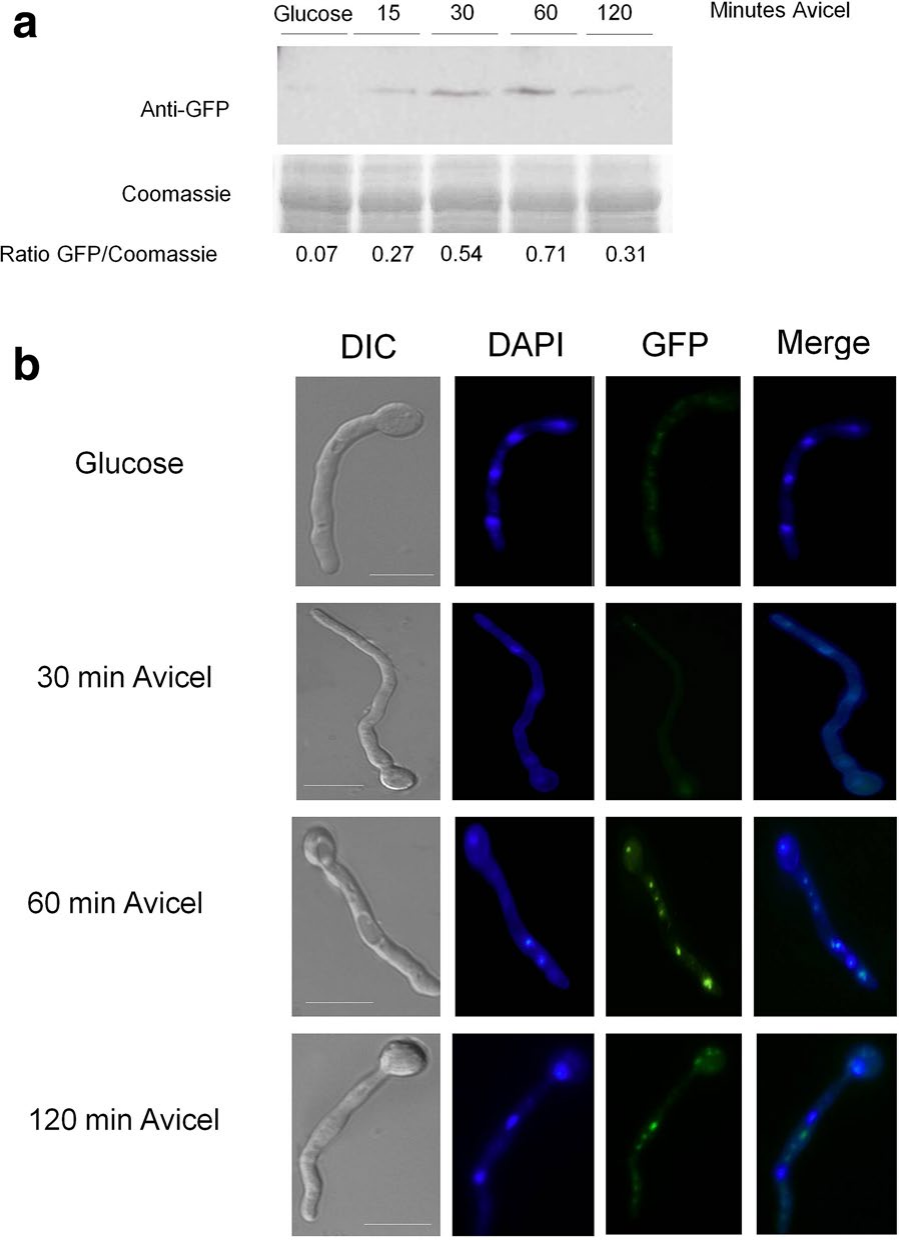
PkaA is involved in the response to carbon starvation. **a** Western blot *pkaA::GFP*. The *pkaA::GFP* strain was grown in minimal media supplemented with 1 % glucose and then transferred to minimal media supplemented with 1 % avicel for the indicated amounts of time. **b** Fluorescence microscopy of PkaA::GFP grown in the same condition described above

**Fig. 6:**
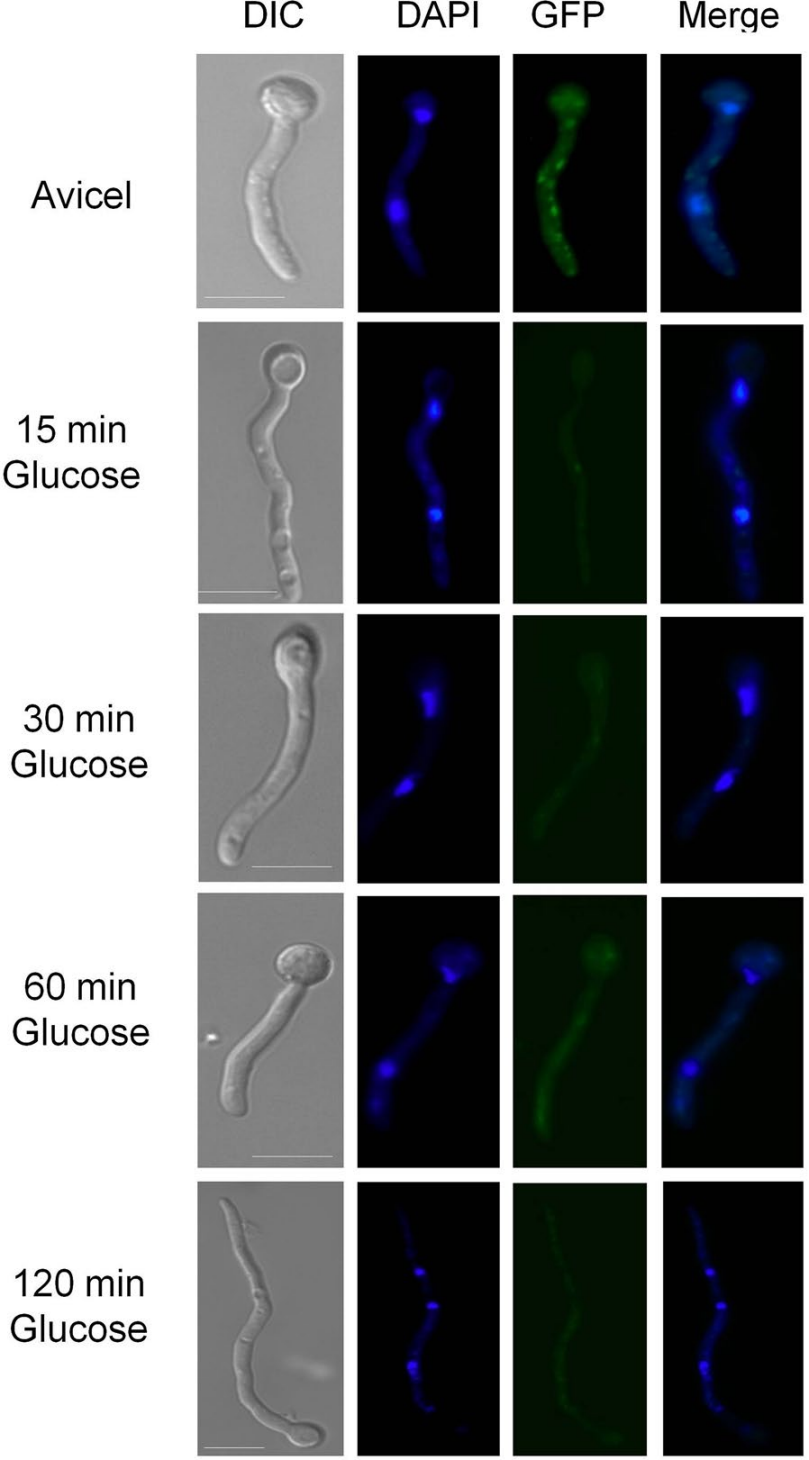
PkaA is involved in the response to carbon starvation. Fluorescence microscopy of *pkaA:GFP* when grown from spores in 1 % avicel for 24 h and then transferred to minimal media supplemented with 1 % glucose as sole carbon source

There is very little information about the translation and/or localization of PkaA in *A. nidulans.* In *S. cerevisiae* there are three homologues of *pkaA*, termed *TPK1*, *TPK2* and *TPK3*, whose expression after carbon source limitation in the stationary phase or in the presence of glycerol was increased [43]. The activity of Tpk1p is controlled by auto-phosphorylation on serine residues. Under starvation or glycerol-rich conditions, Tpk1p is de-phosphorylated [44] and CCR is released. To assess the translation and localization of PkaA in *A. nidulans*, the corresponding PkaA::GFP fusion was constructed. All phenotypes of the PkaA::GFP strain, including growth and conidiation, were essentially identical to the wild-type parent. The PkaA::GFP strain was grown in minimal media supplemented with glucose and then transferred to minimal media supplemented with avicel or without any carbon source for 0, 15, 30, 60 and 120 min. PkaA translation was assessed by Western blot and fluorescence microscopy. Translation of PkaA increased during the first 30 min after transfer to starvation conditions, as revealed by both microscopy and Western blot, whereas after 60 min translation started to decrease (Fig. 4a, c). In accordance, PkaA activity peaked after 30 min in carbon starvation, before dropping again after 2 h (Fig. 4b). Similar results were observed when cellulose was used as a single carbon source (Fig. 5a, b). The opposite was observed under the microscope when growing PkaA::GFP in minimal media supplemented with avicel and then transferred to glucose: fluorescence started to decrease after 15 min in glucose (Fig. 6). These results show that the translation of PkaA increases in carbon starvation conditions.

### PkaA is involved in glucose uptake and glycolysis in *A. nidulans*

**Fig. 7:**
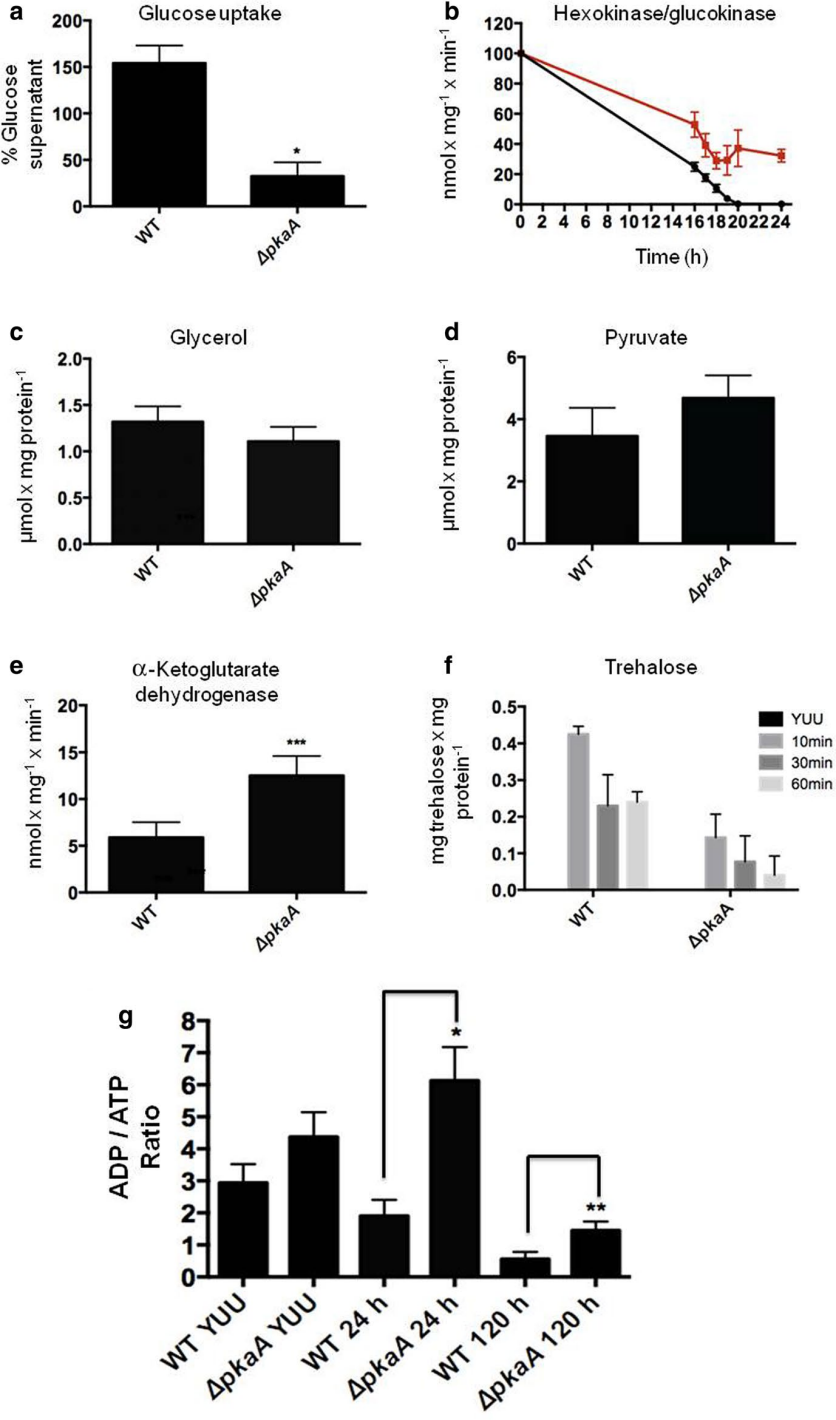
PkaA is involved in glycolysis and controls the expression of genes required for using alternative carbon sources. **a** glucose uptake, **b** hexokinase/glucokinase activity, **c** glycerol levels, **d** pyruvate levels, **e** α-Ketoglutarate dehydrogenase activities and **f** trehalose utilization in the wild-type and Δ*pkaA* strains. Mycelia were grown from spores in complete media and then transferred to minimal media supplemented with glucose for 24 h or to glucose and 1 M sorbitol for 10, 30 and 60 min. **g** Strains were grown in complete media for 24 h and then transferred to minimal media supplemented with 1 % cellulose for 24 and 120 h before the ADP/ATP ratio was measured in mycelia cell extracts. Experiments were carried out in biological triplicates and the statistical significance of *p < 0.05, **p < 0.01 and ***p < 0.001

In mammalian cells, PKA controls the phosphorylation of phosphofructokinase 1 (PFK1), a protein with kinase/phosphatase bi-functional activity. Under glucose limitation, increased levels of cAMP activate PKA which in turn phosphorylates PFK1, hence activating the glycolytic pathway and blocking the gluconeogenesis pathway [45]. This study implicated a role for PkaA in the regulation of CCR and glycolysis in *A. nidulans*. Subsequently, the wild-type and Δ*pkaA* strains were grown in complete medium for 24 h before being transferred to minimal medium supplemented with glucose for 24 h. The ability to take up glucose was then quantified in both strains. The Δ*pkaA* strain showed reduced glucose uptake as after 24-h incubation in glucose there still remained a small amount of glucose in the minimal medium, whereas the wild-type strain consumed all the glucose after 20 h (Fig. 7a). Furthermore, hexokinase/glucokinase activities, the enzymes which convert glucose to glucose-6-phosphate during the first step of glycolysis were reduced in the Δ*pkaA* strain (32.43 nmol mg^−1^ min^−1^) when compared to the wild-type strain (154.07 nmol mg^−1^ min^−1^) (Fig. 7b).

To check if other glucose metabolism-related intermediates were also reduced in the Δ*pkaA* strain, glycerol and pyruvate levels were quantified in both strains after growth in the same conditions as described above. Glycerol and pyruvate levels were similar in the wild-type and Δ*pkaA* strains (Fig. 7c, d). These results suggest that deletion of *pkaA* influences glucose uptake and the first step in glycolysis. The reduction in glucose uptake and subsequent metabolism would lead to the fungus using intracellular reserves. To support this hypothesis, the activity of the mitochondrial enzyme alpha-ketoglutarate dehydrogenase (KGDH) was measured. Indeed, KGDH activity was significantly increased in *ΔpkaA* strain than when compared to the wild-type strain (Fig. 7e). To verify if the *ΔpkaA* strain had reduced ability to accumulate osmolytes such as trehalose, an intracellular storage compound required during fungal spore germination [21, 23, 46–48], the two strains were grown in complete media for 24 h and then transferred to minimal media supplemented with glucose plus 1 M sorbitol. After 10 min of incubation in sorbitol-rich conditions, trehalose levels were increased (0.425 and 0.143 mg trehalose.mg protein^−1^) in both the wild-type and Δ*pkaA* strains, respectively. After 60 min of incubation, trehalose levels were reduced in both strains with the wild-type having 0.24 mg trehalose.mg protein^−1^ and Δ*pkaA* having 0.04 mg trehalose.mg protein^−1^, indicating almost complete use of trehalose in the latter strain (Fig. 7f). In summary, these results indicate that the *ΔpkaA* mutant has reduced glucose uptake and metabolism due to reduced hexokinase activity and, therefore, increased the utilization of intracellular stores such as trehalose, required for maintaining normal glycerol and pyruvate levels, which are generated by gluconeogenesis.

The cells energetic status was then investigated by measuring the intracellular ADP/ATP ratio as glucose metabolism was expected to be decreased in the Δ*pkaA* strain; hence, ATP production should be reduced, increasing the ADP/ATP ratio. Subsequently, the wild-type and Δ*pkaA* strains were grown in complete media for 24 h and then transferred for 24 and 120 h to minimal media supplemented with 1 % avicel. The wild-type strain had an ADP/ATP ratio of 3.0 in complete media and after 24 and 120 h post-transfer to avicel the ratio was reduced to 1.5 and 0.5, respectively, indicating that the glucose released during cellulose degradation was being taken up by the cell. The *ΔpkaA* strain had an ADP/ATP ratio of 4.5 in complete media and after 24 h growth in avicel of 6.0, showing that the glucose released from cellulose degradation is not being completely metabolized which in turn reduced ATP production. After 120 h of growth in avicel, the ratio was reduced to 1.45, because part of the glucose released from cellulose degradation was metabolized. However, the ADP/ATP ratio in the wild-type strain was three times lower than in the Δ*pkaA* strain, further highlighting the reduced ability of this strain to sense, internalize or metabolize glucose (Fig. 7g). Collectively, these results suggest that PkaA influences glycolysis, subsequently affecting CCR and the energetic status of the cell.

## Discussion

The production of bioethanol from plant biomass is economically dependent on the efficiency of hydrolytic enzyme production. To improve the efficiency of hydrolytic enzyme production in filamentous fungi, it is necessary to understand the mechanisms controlling protein synthesis and secretion. This study shows that the deletion of *pkaA* resulted in increased lignocellulolytic enzyme production. Microarray analysis showed that after 8-h incubation in cellulose, cellulase and xylanase gene expression was increased. This increase in gene transcription was not observed in the wild-type strain. Similarly, after 5 days of incubation in cellulose, the Δ*pkaA* strain secreted a higher amount of xylanases and cellulases than the wild-type strain. However, microarray data also showed that after 24 h, the expression of some genes (e.g., *eglA*, *eglB*, *xlnA* and *xlnC*) was similar between both strains. In this study, gene expression was assessed at early time points (8 and 24 h) whereas enzyme activity assays were carried out at a much later time point (5 days). It is likely that lignocellulolytic enzyme activities and regulation of their responding genes, remained high in the Δ*pkaA* strain at all the time points tested, whereas in the wild-type strain, there may be fluctuations in enzyme expression/secretion due to CCR. This hypothesis is further supported by results which showed that lignocellulolytic enzyme activities remained high in the simultaneous presence of an inducing (cellulose/xylan) and repressing (glucose) carbon source in the Δ*pkaA* strain; whereas these enzymes were tightly repressed in the wild-type strain under the same conditions. Furthermore, this work showed that deletion of *pkaA* resulted in severe defects in glucose metabolism. CreA-mediated CCR tightly controls the transcription of lignocellulolytic enzymes [8], favoring the usage of preferred carbon sources such as glucose. Deletion of *pkaA* resulted in the reduced ability of CreA to localize to the nucleus, repressing alternative carbon usage in the presence of glucose. These results indicate that deletion of *pkaA* renders the fungus partially blind to the presence of glucose. In accordance, the genome-wide microarray analyses showed that the deletion of the *pkaA* caused a quicker response to cellulose in the induction of genes encoding cellulases, xylanases, β-glucosidases and β-xylosidases, which are repressed by CreA and are involved in lignocellulose biomass degradation.

Indeed, glucose uptake was reduced in the Δ*pkaA* strain and more interestingly, the activity of the glucokinase/hexokinase which catalyzes the first step in glycolysis is also severely reduced in this deletion mutant. This enzyme phosphorylates glucose which serves as a signal for CreA nuclear localization [4]. In addition, CreA may be unable to locate to the nucleus in the presence of glucose. Thus, deletion of *pkaA* results in a severe defect to the correct uptake and metabolism of glucose, forcing the cell to shift its metabolism to using alternative carbon sources.

Glycolysis results in the production of pyruvate, which can be metabolized by two routes: (1) fermentation, through the pyruvate decarboxylase complex resulting in the production of acetaldehyde which is converted to ethanol [49], or (2) the tricarboxylic acid cycle (TCA). The role of the pyruvate dehydrogenase complex (PDC), which converts pyruvate to acetyl-CoA and directs metabolism towards the TCA cycle is central to determining the fate of pyruvate and reactions triggered by this enzymatic complex are irreversible [50]. Genome-wide microarray analysis showed the down-regulation of genes encoding the PDC, further supporting the hypothesis that the glucose pathway is mis-regulated in *pkaA* deletion mutant. In carbon starvation conditions, the PDC complex is phosphorylated and inactivated by pyruvate dehydrogenase kinases, thus promoting fatty acid utilization [51]. This is in agreement with the microarray results which showed that genes encoding for proteins involved in fatty acid metabolism are up-regulated in the Δ*pkaA* mutant. Fatty acid catabolism has been shown to be important for fungal pathogenesis, secondary metabolite production, metabolism and development [37]. When faced with a nutrient-poor environment, the fungus switches to using other energy sources, one of which is fatty acids. Fungi are able to solely grow on fatty acids [37]. In fungi, fatty acids are first degraded to C4 compounds via the glyoxylate cycle in peroxisomes before further being converted to acetyl-CoA which enters the TCA cycle in the mitochondria [37]. Furthermore, oxidative phosphorylation and ATP production were reduced in the *pkaA* deletion strain, supporting the hypothesis that the cell is exhibiting a starvation response in the presence of glucose. This also explains the early and constant up-regulation of genes encoding lignocellulolytic enzymes as these have been proposed to have a “scavenger role” under carbon starvation conditions in *A. niger* and *T. reesei* [52, 53].

**Fig. 8:**
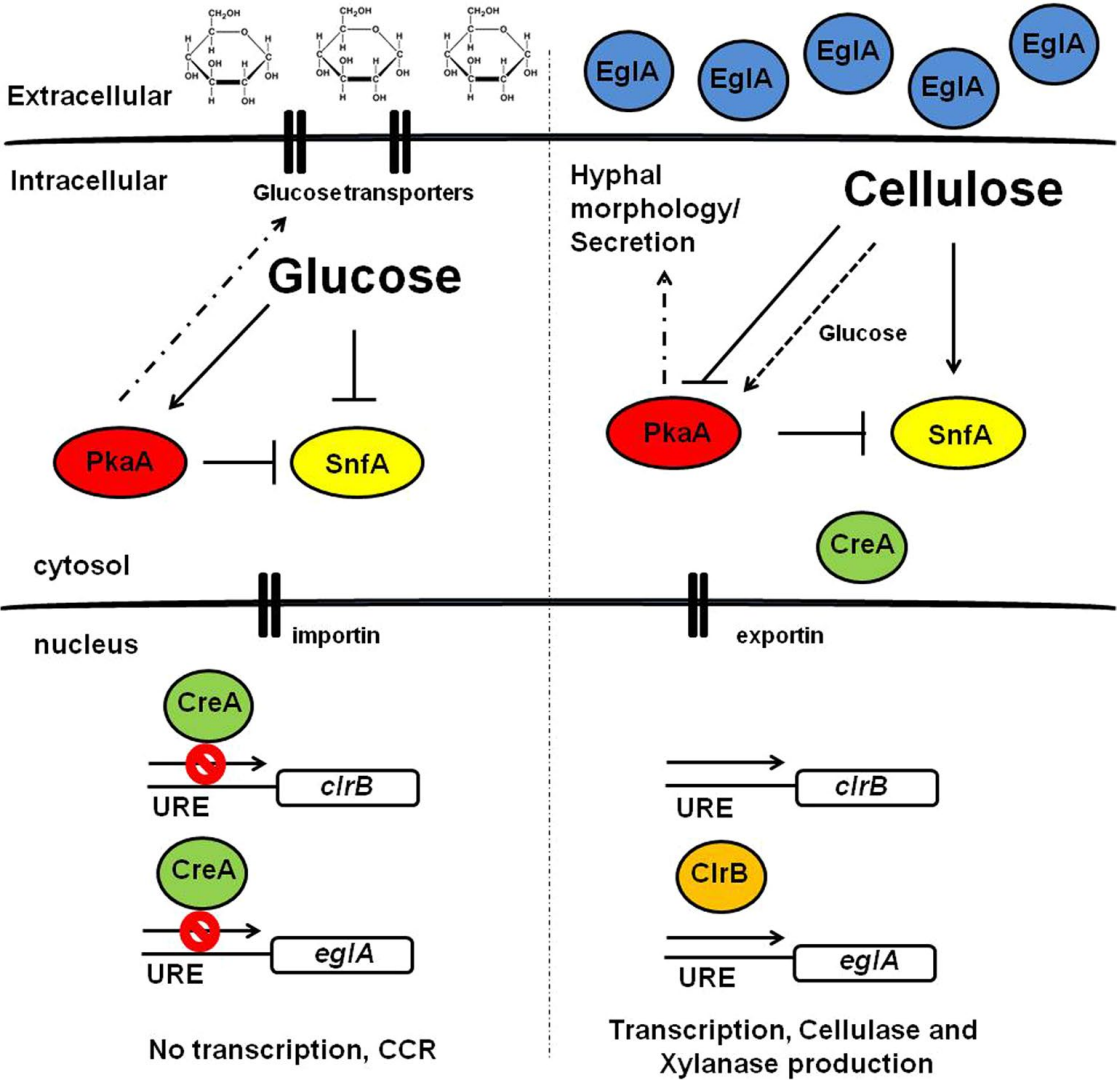
A possible model for the interaction between *A. nidulans* PkaA and SnfA during carbon catabolite repression (CCR) repressing (glucose) and de-repressing (cellulose) conditions. In the presence of glucose, *A. nidulans* PkaA is activated and represses SnfA. CreA is transported into the nucleus via importins where it represses either directly or indirectly the expression of cellulase-encoding genes (e.g., *eglA*) and their corresponding positive regulators (e.g., *clrB*). In the presence of cellulose, PkaA is inactive whereas SnfA is activated and probably mediates the phosphorylation and re-localization of CreA into the cytoplasm, resulting in cellulose gene de-repression. It is unknown whether SnfA is transported into the nucleus or if there is another intermediary protein that is phosphorylated by SnfA and which is responsible for CreA removal from the nucleus. The role played by nuclear importins/exportins during these processes also remains unknown. PkaA activity affects hyphal morphology, protein secretion and glucose transport

The SnfA kinase complex, which controls alternative carbon usage, is key for the de-repression of CreA-mediated CCR and hydrolytic enzyme transcription [4]. Extracellular enzymatic levels do not change in the *snfA* deletion strain under repressing and de-repressing conditions (this strain appears to secrete a basal level of lignocellulolytic enzymes), whereas in the *ΔpkaA ΔsnfA* strain cellulase levels were increased. This shows that PkaA and SnfA genetically interact, but the details of this interaction remain subject to further investigation. Nonetheless, it would appear that PkaA and SnfA have opposing functions, i.e., PkaA is important for glucose-mediated-catabolite repression while SnfA is important for catabolite de-repression. It remains to be investigated the details of the interaction between these protein kinases (for a model, see Fig. 8). In the presence of glucose, PkaA is activated and regulates indirectly the nuclear localization of CreA. This work showed that PkaA controls the activity of hexokinase which catalyzes the first step in glycolysis and phosphorylates glucose. Phosphorylated glucose has been shown to be (one of) the signal(s) for CreA localizing to the nucleus in *A. nidulans* [8]. Furthermore, this work showed that PkaA is involved in the regulation of SnfA by repressing it. SnfA becomes inactive in glucose-rich conditions as it is required for the use of alternative carbon sources such as cellulose [39, 40, 54, 55]. In the presence of non-glucose, complex carbon sources (e.g., cellulose), PkaA is inactive, SnfA becomes activated and subsequently CreA localizes to the cytoplasm.

There is a significant lack of information on the components of the signaling pathways in which both PkaA and SnfA are involved and Fig. 8 only shows a very preliminary diagram on how both kinases are interacting and their involvement in CreA cellular localization. Furthermore, the level of PkaA (and probably SnfA) activity is also likely subject to fluctuations and other regulations as this work showed that it is up-regulated in cellulose the first 30 min after transfer from glucose. The details of the respective pathways remain subject to investigation.

Beyond transcriptional regulation the production of enzymes by filamentous fungi is influenced by hyphal morphology and different morphological forms can have a significant effect on enzyme production within a bioreactor [56–58]. The Δ*pkaA* strain exhibited reduced growth and morphological alterations in the presence of glucose as a single carbon source. Although we did not identify in detail the causes of these morphological alterations, the Δ*pkaA* mutant showed the increased induction of the *stuA* transcription factor, which is involved in spatial conidial formation, cleistothecia, Hulle cell formation and germination [23]. An increase in the expression of genes encoding transcription factors involved in controlling development could explain why the *pkaA* deletion strain exhibited an increase in the number of branches and tip formation when grown in complete media and in minimal media supplemented with glucose or CMC. No difference in the localization of GFP::SynA, a protein which localizes to secretory vesicles, was observed in the wild-type and Δ*pkaA* strains when examined by fluorescent microscopy. However, Western blot analysis showed a reduction in GFP::SynA after 5 days of growth in cellulose in the wild-type strain but not in the Δ*pkaA* strain. Furthermore, microarray analysis identified several genes encoding putative components of secretion to be up-regulated during the first 8 h of incubation in glucose; after 24 h expression levels of these genes were similar to those in the wild-type strain. Again, these results indicate that protein secretion remains high in the Δ*pkaA* strain throughout all the time points tested here, whereas in the wild-type strain they appear to be subject to fluctuations. These results suggest that the increase in enzyme secretion in *pkaA* deletion strain was due to PkaA playing a role in protein secretion and in hyphal morphology. An increase in the number of hyphal tips from which proteins are secreted could contribute to the increased secretion of hydrolytic enzymes by the Δ*pkaA* strain.

## Conclusion

In summary, PkaA is involved in controlling the cellular metabolic and energetic status. PkaA is directly involved in glycolysis and deletion of this protein kinase resulted in CCR mis-function, forcing the cell into a state of starvation, where hydrolytic enzyme secretion is increased, expression of genes encoding mitochondrial components as well as genes involved in fatty acid utilization were up-regulated. This works implies that PkaA regulates the secretion of enzymes required for plant biomass degradation. The absence of PkaA leads to the cell secreting higher amounts of hydrolytic enzymes.

This work further contributes to unraveling the metabolic pathways governing different states of the cell. In addition, this study highlights how fungal morphology can impact on enzyme secretion. Furthermore, deletion of *pkaA* resulted in reduced glucose uptake and a defect in glycolysis, which as a consequence, reduces CCR, resulting in hydrolytic enzyme induction in the presence of inducer molecules. Deletion of *pkaA* also results in the inability to perform glycolysis which puts the cell in a state of carbon starvation. This work shows that PkaA is specifically induced in carbon starvation conditions, as has also been reported in other organisms. Together, this study identified various roles in carbon metabolism for PkaA within the cell and shows how a tightly interconnected metabolic network governs lignocellulolytic enzyme production and secretion. This work provides a basis for further research which aims at elucidating cellular regulatory pathways which can be useful for the further engineering of fungal strains, which are highly efficient in protein secretion with the aim to use these enzymes in various industrial processes. Up to this point, the direct and indirect targets of PkaA are unknown and uncovering these proteins could provide a platform for the engineering of fungal strains with improved plant biomass degradation capabilities.

## Methods

### Strains and culture conditions

The two *A. nidulans* strains TN02A3 (*pyrG89*; *pyroA4*; *nkuA::argB*) and A4 were used as reference strains (wild type). The *ΔpkaA* and *ΔsnfA* null mutants used in this work were obtained from the protein kinase deletion collection [59] and are publicly available at the Fungal Genetics Stock Center (http://www.fgsc.net). A list of all strains used in this study is found in Table 4. All strains were grown at 37 °C in either liquid (without agar) or solid (with 20 g/l agar) minimal medium [MM: 1 % (w/v) carbon source, 50 mL of a 20× salt solution (120 g/l NaNO_3_, 10.4 g/l KCl, 30 g/l KH_2_PO_4_, 10.4 g/l MgSO_4_), 1 mL of 5× trace elements [22.0 g/l ZnSO_4_, 11 g/l boric acid, 5 g/l MnCl_2_, 5 g/l FeSO_4_, 1.6 g/l CoCl_2_, 1.6 g/l CuSO_4_, 1.1 g/l (NH_4_)_2_MoO_4_, 50 g/l ethylenediaminetetraacetic acid (EDTA)], pH 6.5 or in liquid complete medium complete [2 % w/v glucose, 0.5 % w/v yeast extract, trace elements (same as described above)]. Depending on the auxotrophy of the strain, uridine (1.2 g/l), uracil (1.2 g/l) or pyridoxine (0.005 mg/μL) were added.

**Table 4:** *A. nidulans* strains used in this study

Strain	Genotype	References
TN02A3	*pyroA4 pyrG*89; *chaA*1; Δ*nKuA*::*argB*	[73]
R21	*pabaA*1; *yA2*	FGSC
Δ*pkaA*	*pyrG*89; *wA3*; *argB*2; Δ*nkuAku*70::*argB pyroA*4; *sE15* *nirA*14 *chaA*1 fwA1; Δ*pkaA*::*pyrG* ^+^	[59, 74]
Δ*snfA*	*pyrG*89; *wA3*; *argB*2; Δ*nkuAku*70::*argB pyroA*4; *sE15 nirA14 chaA1 fwA1*; Δ*snfA*::*pyrG* ^+^	[73, 74]
Δ*snfA* PABA^−^	*pyrG89; wA3; argB2; ΔnkuAku70::argB pyroA4; sE15 nirA14 chaA1 fwA1; pabaA1; ΔsnfA::pyrG* ^+^	[8]
Δ*pkA* Δ*snfA*	*pyrG89; wA3; argB2; ΔnkuAku70::argB pyroA4; sE15 nirA14 chaA1 fwA1; pabaA1; ΔpkaA::pyrG* ^+^ *; ΔsnfA::pyrG* ^+^	This study
GFP::SynA PIRO^−^	*pyrG89, GFP::synA::pyrGAf, nkuA::bar, pyroA4*	[41]
GFP::SynA PABA^−^	*pyrG89, gfp::synA::pyrGAf, nkuA::bar, pyroA4 pabaA1*	This study
GFP::SynA Δ*pkA*	*pyrG89; pabaA1; GFP::synA::pyrGAf, nkuA::bar, pyroA4; ΔpkA::pyroA4*	This study
PkaA::GFP	*pyroA4; pyrG89; chaA1; ΔnKuA::argB; pkaA::GFP pyroA4*	This study

The genotypes of each strain are also shown

### Strain construction

The construction of the Pka::GFP strain was performed according to Colot et al. (2006) [60]. Standard molecular techniques were performed according to Sambrook and Russel [61]. The *pkaA* 5′ and 3′ untranscribed regions (UTR), ORF (open reading frame) plus the *pkaA* gene (minus the stop codon), *gfp* gene and spacer region and the *pyrG* gene were co-transformed into *S. cerevisiae* SC9721 strain (*MATα his3*-*Δ200 URA3*-*52 leu2Δ1 lys2Δ202 trp1Δ63*) obtained from the Fungal Genetic Stock Center (FGSC). Primer sequences are described in Table 5. The *pkaA* 5′UTR and ORF were amplified using the primers “pRS426-5′ PKA UTR F’” and “PKA Spacer GFP R”; the *gfp* gene was amplified using primers “Spacer GFP Fw” and “GFP Ve 3 Afu Rv”; the *pyrG* fragment was generated using primers “GFP-PyrG Fw” and “Afu PyrG Rv FGSC” and the *pkaA* 3′ UTR region was amplified using primers “PyrG 3 UTR PKA F” and “PKA 3 UTR-pRS426″. Homologous recombination within *S. cerevisiae* created the construct, which was subsequently amplified from pooled *S. cerevisiae* DNA, and 20 μg was subsequently transformed into TN02a3 according to Osmani et al. (1987) [62]. Transformants were selected via their ability to grow on solid MM supplemented with pyridoxine in the absence of uridine and uracil.

**Table 5:** List of the primer pair used in this work

Primer	Sequence
pRS426-5′ PKA UTR F	TAACGCCAGGGTTTTCCCAGTCACGACGTTCTGAAGCCCGATACAACC
PKA Spacer GFP R	AAAGTTCTTCTCCTTTACTCATTCCCCGTGTTCCGAAATCGGGGAACAGGTGACCG
PyrG 3 UTR PKA F	AAGAGCATTGTTTGAGGCGAATTCACCCTCTAACGAGTGATG
PKA 3 UTR-pRS426 R	GCGGTTAACAATTTCTCTCTGGAAACAGCTCTAAGGCAGGCAGTTCTCG
GFP PyrG FW	GCATGCAAGCTTGGCGTATTCTGTCTGAGAGGAGGC
Afu PyrG RV FGSC	GAGCAGCGTAGATGCCTCGACC
Spacer GFP FW	GGAACACGGGGAATGAGTAAAGGAGAAGAACT
GFP Ve 3 Afu RV	CTCAGACAGAATACGCCAAGCTTG
pRS426-5′ snfA UTR F	GTAACGCCAGGGTTTTCCCAGTCACGACGTGGAGATGGAAGTCGAAAGG
CreA GFP	ATAGACATGCCGTCACATGG
Afu pyrG pCDS60 R	GAGCAGCGTAGATGCCTCGACC
F_pkA_checkinsert_A	ATGGGTCCGACACCAAGA

Homologous integration was confirmed by PCR using a forward primer that anneals out of the recombination locus (F_pka_checkinsert_A) and a reverse primer (GFP Ve 3 Afu R) that anneals at the end of the GFP gene (Table 5). The *∆pkaA ∆snfA* and GFP::*synA* Δ*pkaA* strains were generated by sexually crossing the parental strains and the genotype of the double mutant was confirmed by PCR (Table 5) and in case of the GFP construction, by microscopy. The deletion of *pkaA* and *snfA* were confirmed by PCR using primers pRS426-5′ PKA UTR F and pRS426-5′ snfA UTR F, respectively, with Afu PyrG RV FGSC as the reverse primer for both constructions.

### Microarray analysis

Initially 1 × 10^7^ conidia *A. nidulans* were inoculated in complete media at 37 °C in a rotatory shaker (180 rpm) for 24 h. Subsequently, mycelia were washed with sterile water and then incubated in minimal media supplemented with 1 % cellulose at 37 °C for an additional 8 and 24 h. At each step the mycelia from three biological replicates were collected by vacuum filtration and immediately frozen in liquid nitrogen. Agilent custom-designed oligonucleotides arrays [63] were used to identify the transcriptional differences during growth on complete media (Cy3 reference) and cellulose (Cy5) for the wild-type and Δ*pkaA* strains. Total RNA was extracted and RNA integrity confirmed as described in the section “RNA extraction”. Array hybridization and data analysis were performed according to De Assis et al. (2015) [50]. The dataset was deposited in the Gene Expression Omnibus (http://www.ncbi.nlm.nih.gov/geo/query/acc.cgi?acc=GSE70917) under the number GSE70917. Genes were determined as differentially expressed between carbon sources by applying a *t* test (*p* < 0.01) performed within the Mev software [64]. The functional profile and identification of overrepresented GO terms within the differentially expressed gene sets from each strain under the two nutritional conditions were identified using the GO Slim mapper (http://www.aspergillusgenome.org/cgi-bin/GO/goTermMapper) and FunCat (http://mips.helmholtz-muenchen.de/funcatDB/).

### RNA extraction

Mycelia were harvested by vacuum filtration and immediately frozen in liquid nitrogen. Mycelia were ground to a fine powder under liquid nitrogen and total RNA was extracted using TRIZOL, according to manufacturer’s instructions (Invitrogen), before being treated with RNAse-free DNAse (Promega) and purified with the RNeasy^®^ Mini Kit, according to manufacturer’s instructions (Qiagen). RNA integrity was confirmed using the Bioanalyser Nano Kit (Agilent Technologies) and the Agilent Bioanalyser 2100, considering RIM value 8.0 as the RNA quality threshold. The SuperScript III First Strand Synthesis system (Invitrogen) and oligo(dT) primers were used for cDNA synthesis according to manufacturer’s instructions. All the primer sequences used in this work are described in the Additional file 5: Table S4.

### Xylanase and cellulase assays

Xylanase (endo-1,4-β-xylanase) and cellulase (endo-1,4-β-glucanase) assays were performed using Azo-Xylan (Birchwood) and Azo-CM-Cellulose (both from Megazyme International, Bray, Ireland) as substrates, according to manufacturer’s instructions. All enzyme assays were carried out on the supernatants of biological triplicates. Technical triplicates were carried out on each biological replicate.

### β-Glucosidase and β-xylosidase assays

β-Glucosidase (BGL) and β-xylosidase (BXL) activities were measured in 20 μL culture supernatants. ρ-Nitro phenyl glucopyranoside (ρ-PNG) and 4-β-D-xylopyranoside (ρ-PNG) were used as substrates for BGL and BXL activities in 50 mM buffer citrate pH 6.0 as previously described [65]. Enzyme activities were calculated using the slope of the linear curve generated during 30 min of reaction at 37 °C. All enzyme assays were carried out on the supernatants of biological triplicates. Technical triplicates were then carried out on each biological replicate.

### Fluorescence microscopy

Strains were inoculated in 3 mL MM supplemented with 1 % glucose, cellulose or CMC in a small Petri dish with a cover slip and incubated for 16 h at 22 °C. For the transfer experiments, coverslips were washed three times with water and then transferred to minimal media supplemented with a different carbon source for 5 h.

### Secretome and cellulase assay on plate

Mycelia were grown from 10^7^ spores in 50 mL media in the specified conditions. Culture supernatants were separated from the mycelia and centrifuged at 1500 g at 4 °C for 5 min. Supernatants (40 mL) were transferred to new clean tubes and freeze-dried before being re-suspended in 3 mL buffer containing 50 mM Tris–HCl pH 7.0, 1 mM DTT and protease inhibitors (EDTA-free Complete mini, Roche). About 20 μL of the re-suspended supernatants were run on a 10 % SDS-PAGE gel. The gel was then silver stained as described previously [66]. The cellulase assay on plate was carried out as previously described [67, 68].

### Western blots

Mycelia were grown from spores in the specified conditions before being harvested and ground to a fine powder under liquid nitrogen. Mycelial powder were re-suspended in extraction buffer [50 mM Tris–HCl pH 7.0, 50 mM NaF, 1 mM Na_3_VO_4_, 1 mM DTT, phosphatase inhibitor cocktail P0044 (Sigma) and the complete mini EDTA-free protease inhibitor cocktail (Roche)], prior to centrifugation for 5 min at 14000×*g*. The concentration of the protein extracts was measured using the Bio-Rad Bradford protein assay, according to manufacturer’s instructions. Proteins were precipitated with 0.25 M NaOH and 1 % β-mercaptoethanol and incubated on ice for 10 min before trichloroacetic acid was added to a final concentration of 6 % for 10 min on ice. Samples were pelleted by centrifugation for 5 min at 4°, maximum speed. Pellets were re-suspended in electrophoresis Bolt (Life Technologies) LDS sample buffer and reducing agent (according to manufacturer’s instructions). Samples were run on Bolt^®^ 4–12 % Bis–Tris Gels (Life Technologies) before proteins were transferred to membranes with the iBlot^®^ 2 Gel transfer device, according to manufacturer’s instructions. Membranes were incubated with a 1:10,000 dilution of anti-GFP antibody (AbCam ab290) overnight at 4 °C. The next day membranes were washed and incubated with a 1:5000 dilution of secondary antibody Rabbit (Cell Signaling). Proteins were detected using the Super Signal West Pico (Thermo Scientific) chemiluminescent substrate according to manufacturer’s instructions.

### Glucose uptake

Mycelia were grown from 3 × 10^6^ spores in 30 mL complete media for 24 h at 37 °C in a rotary shaker (180 rpm). Mycelia were then transferred to minimal medium supplemented with 1 % glucose as a carbon source for an additional 24 h. The amount of free glucose remaining in the culture supernatants was measured using the Glucose GOD-PAP Liquid Stable Mono-reagent kit (LaborLab Laboratories Ltd. Guarulhos, São Paulo, Brazil), according to the manufacturer’s instructions. Glucose uptake was calculated via determining the difference in glucose present in the initial media and at different time points during the 24-h incubation in MM.

### Hexokinase/glucokinase activities

Proteins were extracted from mycelia grown in the specified conditions and protein content was measured as described in the section “Western blots”. The hexokinase/glucokinase activity was measured in protein extracts according to [69] with some modifications. 3 μg of total protein extract was mixed with reaction buffer (50 mM Hepes pH 7.5, 50 mM KCl, 5 mM MgCl_2_, 2 mM ATP, 1 mM phosphoenolpyruvate, 0.4 mM NADH, 5 U pyruvate kinase, 15 U lactate dehydrogenase) to a final volume of 200 μL. Samples were first incubated for 30 min at 37 °C to stabilize the reactions and get an even reaction curve. Enzymatic reactions were triggered with the addition of 100 mM glucose and the decline in absorbance at 340 nm was measured during 15 min at 37 °C in the SpectraMax I3 spectrometer (molecular devices).

### Pyruvate and glycerol measurement

Proteins were extracted from mycelia grown in the specified conditions and protein content was measured as described in the section “Western blots”. Glycerol and pyruvate concentrations were measured in 5 and 10 μg, respectively, of total extracted protein as described previously [50].

### Alpha-ketoglutarate assay

Proteins were extracted from mycelia grown in the specified conditions and protein content was measured as described in the section “Western blots”. Alpha-ketoglutarate activity was measured in 30 μg of total extracted protein as described in [70–72] with modifications. The reaction buffer consisted of 100 mM Tris–HCl pH 7.0, 5 mM 2-mercaptoethanol, 1 mM MgCl_2_, 2 mM thiamine pyrophosphate (TPP), 5 mM alpha-ketoglutarate acid disodium salt, 1 mM NAD^+^ and 0.2 mM Coenzyme A. The reduced form of NADH was measured at 340 nm in the SpectraMax I3 spectrometer (molecular devices).

### PKA Activity

Proteins were extracted from mycelia grown in the specified conditions and protein content was measured as described in the section “Western blots”. PKA activity was measured using PepTag Assay Non-radioactive Detection of cAMP-dependent Protein Kinase assay Ref. V5340 (Promega) according to manufacturer’s instructions.

### Trehalose assay

Proteins were extracted from mycelia grown in the specified conditions and protein content was measured as described in the section “Western blots”. The trehalose content was measured in 10–20 μg protein using the Trehalose Assay kit K-TREH 11/12 (Megazyme) according to manufacturer’s instructions. A standard curve of 0–4 μg trehalose dehydrate was also prepared.

### ADP/ATP ratio

Proteins were extracted from mycelia grown in the specified conditions and protein content was measured as described in the section “Western blots”. The ADP/ATP ratio was measured in 10 μg of total protein extract using the ADP/ATP ratio assay kit MAK135 (Sigma) following the manufacturer’s instructions. Luminescence was read in the SpectraMax I3 spectrometer (Molecular Devices).

### Statistical analysis

Statistical analyses were performed for all reactions of three biological replicates using a one-tailed *t* test (Prism, GraphPad) with *p < 0 0.05, **p < 0.01 and ***p < 0.001.

## Additional files

10.1186/s13068-015-0401-1 List of CAZymes.
10.1186/s13068-015-0401-1 MIPS FunCat 8 h.
10.1186/s13068-015-0401-1 MIPS FunCat 24 h.
10.1186/s13068-015-0401-1 Deletion of pkaA results in a severe growth defect. Strains ΔpkaA, ΔacyA, R21, ΔsnfA and ΔpkaA snfA were grown in a concentration from 105 to 102 (left to right) from spores on different carbon sources [YUU (Complete media), Glu (glucose), Casa (casaminoacids), Gly (glycerol), Xyl (Xylose), Fru (fructose) and Trybut (trybutirin)]. Figure S2. Expression of GFP::SynA. A. Microscopy picture (GFP) of GFP::SynA grown for 16 h at 22 °C in 1 % cellulose. B. Fluorescence in mycelia which were grown from spores in minimal media supplemented with 1 % cellulose at 22 °C for 16 h. Fluorescence was then assessed using ImageJ freeware. An average of 50 pictures were taken and evaluated for each strain. Figure S3. The ΔpkaA strain secretes more proteins. A) Cellulase secretion in different strains. Strains were grown (upper row) for 48 h on minimal media supplemented with 1 % CMC (carboxymethylcellulose) as sole carbon source. Plates were then strained with congo red (lower row) and the halo/mycelia was measured. B) Secretion of proteins by the wild-type and ΔpkaA strains. Strains were grown in triplicates from spores in complete media for 16 h and then transferred to minimal media supplemented with 1 % cellulose for 5 days. Culture supernatants were harvested, dried and re-suspended before proteins were run on a SDS-PAGE gel and silver stained. Arrows indicate proteins secreted by the pkaA mutant but not by the wild-type strain.
10.1186/s13068-015-0401-1 List of the primer pair used in this work.

## Abbreviations

AA: auxiliary activities
ADP: adenosine diphosphate
ATP: adenosine triphosphate
BGL: β-glucosidase
BXL: β-xylosidase
cAMP: cyclic adenosine monophosphate
CAZy: carbohydrate-active enzymes
CBH: cellobiohydrolases
CCR: carbon catabolite repression
CE: carbohydrate esterases
CMC: carboxymethyl cellulose
DTT: dithiothreitol
EDTA: ethylenediaminetetraacetic acid
FAD: flavin adenine dinucleotide
FMN: flavin mononucleotide
GFP: green fluorescent protein
GH: glycoside hydrolases
GO: gene ontology
GPCR: G protein-coupled receptor
GT: glycosyltransferases
KCl: potassium chloride
KGDH: alpha-ketoglutarate dehydrogenase
MgCl_2_: magnesium chloride
MM: minimal media
Na_3_VO_4_: sodium orthovanadate
NADH: nicotinamide adenine dinucleotide reduced form
NaF: sodium fluoride
ORF: open read frame
PDC: pyruvate dehydrogenase complex
PKA: cAMP-dependent protein kinase catalytic subunit A
PL: polysaccharide lyases
TCA: tricarboxylic acid cycle
TPP: thiamine pyrophosphate
V-SNARE: synaptobrevin protein involved in the secretion pathway
YG: complete media (yeast extract and glucose)
ρ-PNG: ρ-nitro phenyl glucopyranoside
ρ-PNX: 4-β-d-xylopyranoside
