# Supplementary material for: Aspergillus nidulans protein kinase A plays an important role in cellulase production
Source: Biotechnol Biofuels. 2015 Dec 18;8:213. doi: 10.1186/s13068-015-0401-1 (PMC4683954; doi:10.1186/s13068-015-0401-1)
Supplement: Supplementary file 4 — 10.1186/s13068-015-0401-1 Deletion of pkaA results in a severe growth defect. Strains ΔpkaA, ΔacyA, R21, ΔsnfA and ΔpkaA snfA were grown in a concentration from 105 to 102 (left to right) from spores on different carbon sources [YUU (Complete media), Glu (glucose), Casa (casaminoacids), Gly (glycerol), Xyl (Xylose), Fru (fructose) and Trybut (trybutirin)]. Figure S2. Expression of GFP::SynA. A. Microscopy picture (GFP) of GFP::SynA grown for 16 h at 22 °C in 1 % cellulose. B. Fluorescence in mycelia which were grown from spores in minimal media supplemented with 1 % cellulose at 22 °C for 16 h. Fluorescence was then assessed using ImageJ freeware. An average of 50 pictures were taken and evaluated for each strain. Figure S3. The ΔpkaA strain secretes more proteins. A) Cellulase secretion in different strains. Strains were grown (upper row) for 48 h on minimal media supplemented with 1 % CMC (carboxymethylcellulose) as sole carbon source. Plates were then strained with congo red (lower row) and the halo/mycelia was measured. B) Secretion of proteins by the wild-type and ΔpkaA strains. Strains were grown in triplicates from spores in complete media for 16 h and then transferred to minimal media supplemented with 1 % cellulose for 5 days. Culture supernatants were harvested, dried and re-suspended before proteins were run on a SDS-PAGE gel and silver stained. Arrows indicate proteins secreted by the pkaA mutant but not by the wild-type strain. [file 13068_2015_401_MOESM5_ESM.pdf]

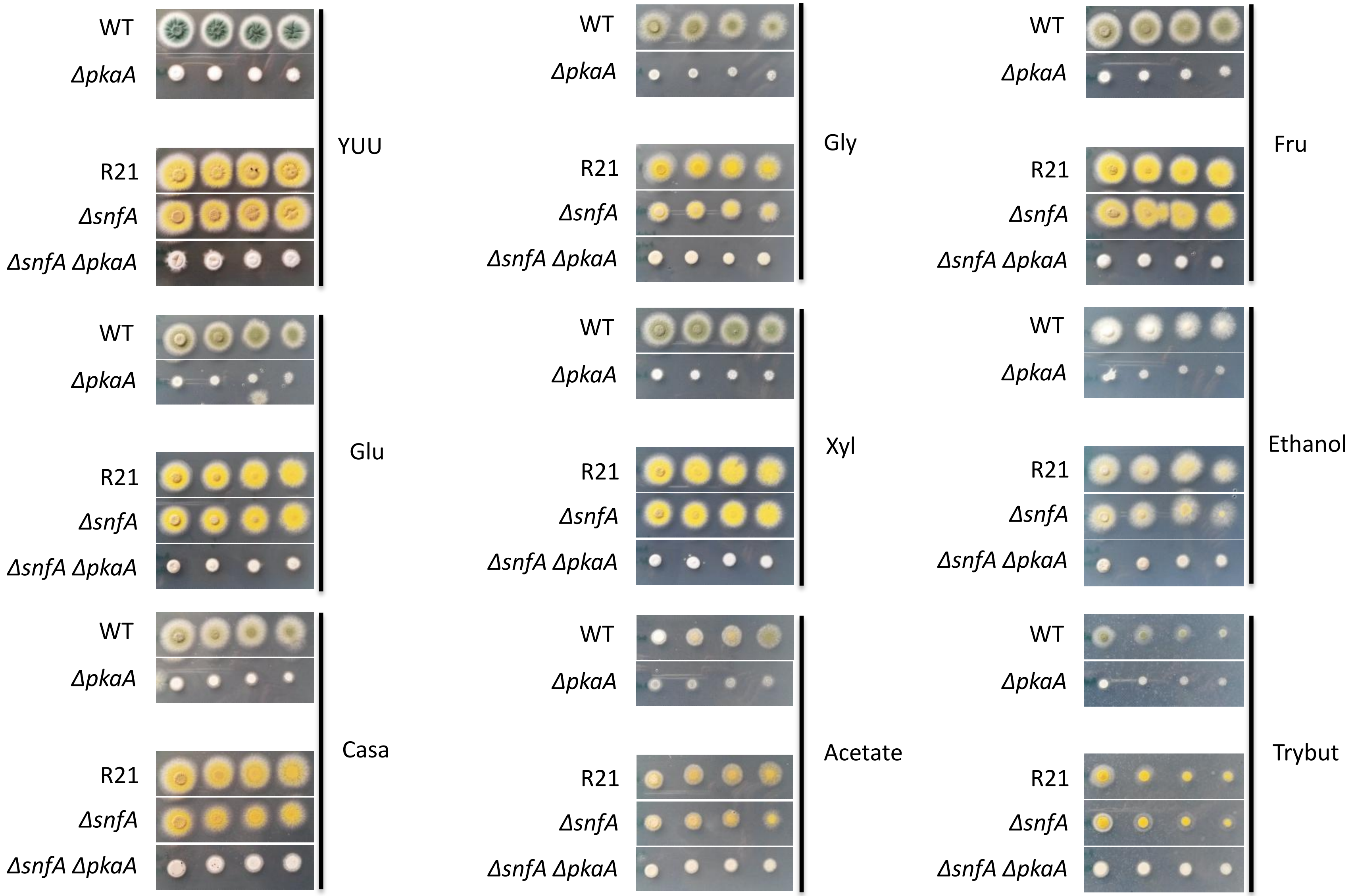

Figure S1. Deletion of *pkaA* results in a severe growth defect. Strains *ΔpkaA,* wild-type (R21), *ΔsnfA and ΔpkaA snfA* were grown in a concentration from 10^5 to 10^2 (left to right) from spores on different carbon sources [YUU (Complete media), Glu (glucose), Casa (casaminoacids), Gly (glycerol), Xyl (Xylose), Fru (fructose) and Trybut (trybutirin)].

A.

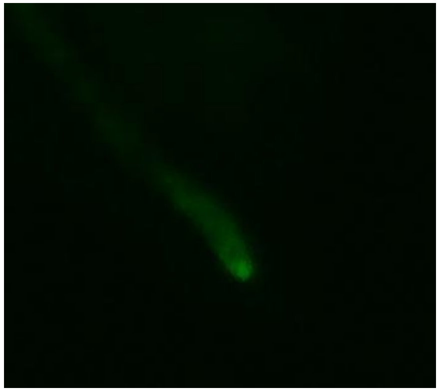

B.

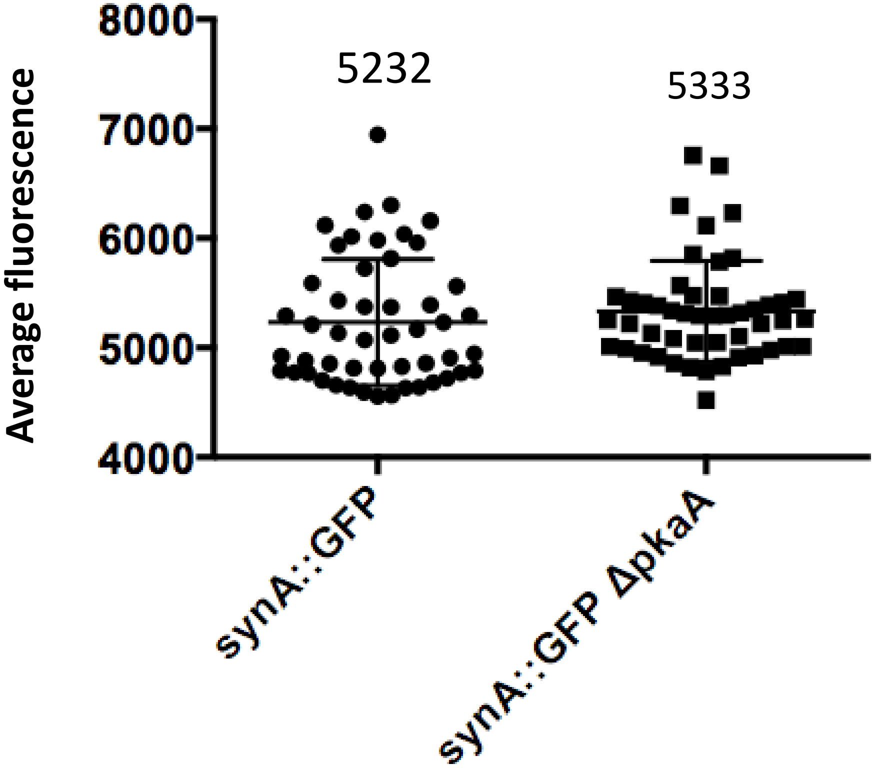

Figure S2. Expression of synA::GFP. **A.** Microscopy picture (GFP) of synA::GFP grown for 16 h at 22°C in 1% cellulose. **B**. Fluorescence of mycelia which were grown from spores in minimal media supplemented with 1% cellulose at 22˚C for 16 h. Fluorescence was then assessed using ImageJ freeware. An average of 50 pictures were taken and evaluated for each strain.

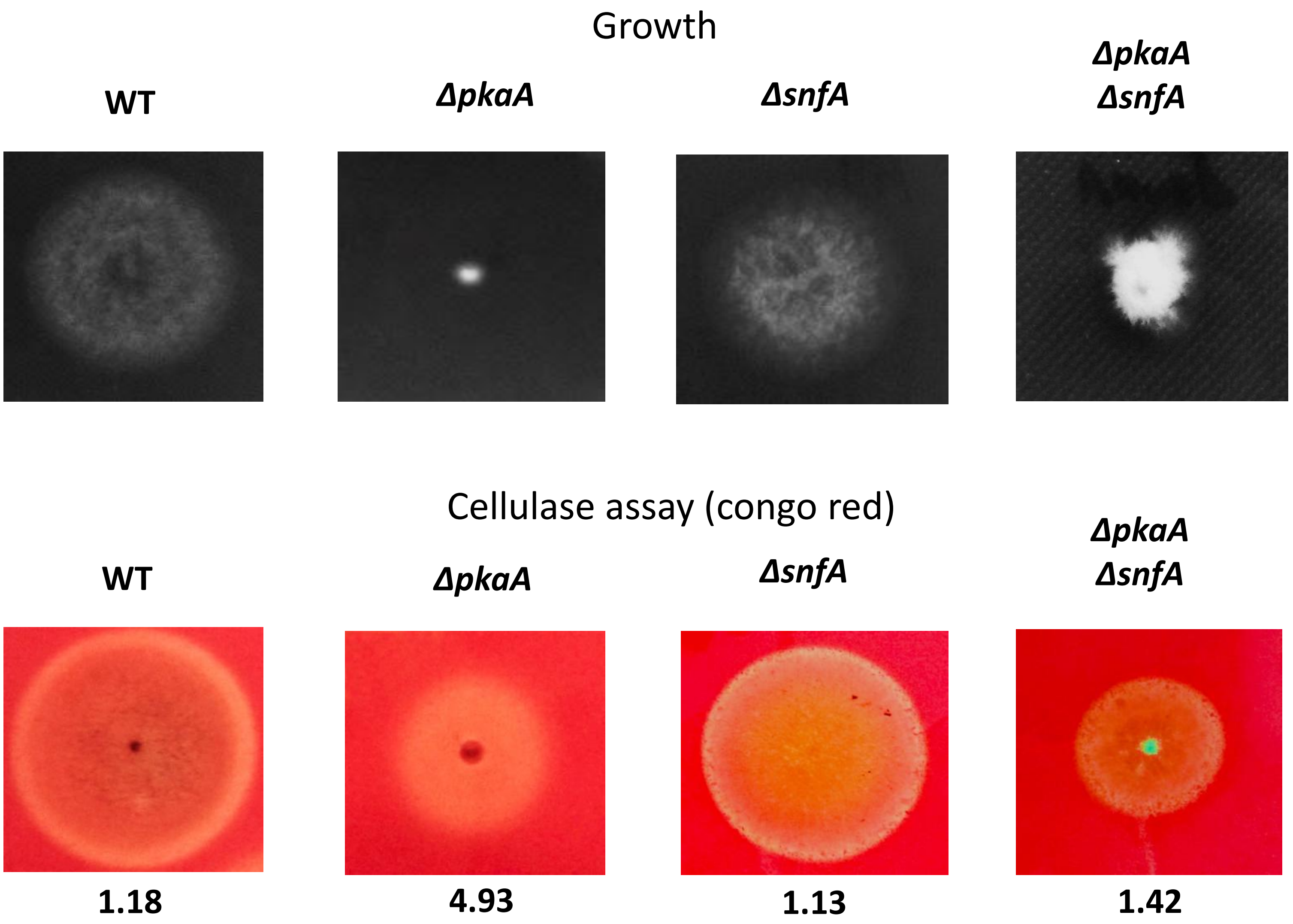

Figure S3. A) Cellulase secretion in different strains. Strains were grown (upper row) for 48 h on minimal media supplemented with 1% CMC (carboxymethylcellulose) as sole carbon source. Plates were then strained with congo red (lower row) and the halo/mycelia was measured.

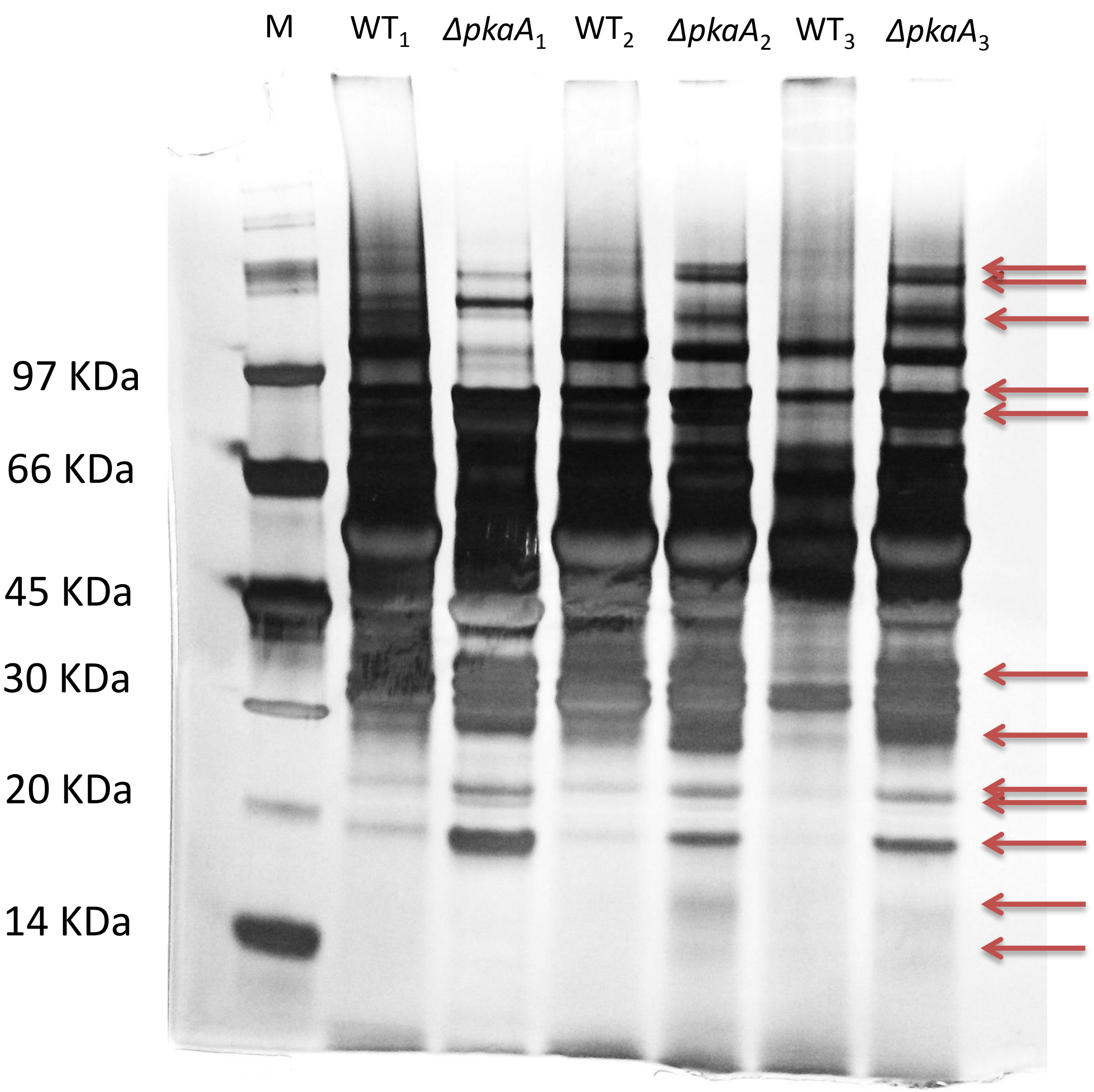

Figure S3. B) Secretion of proteins by the wild-type and Δ*pkaA* strains. Strains were grown in triplicates from spores in complete media for 16 h and then transferred to minimal media supplemented with 1% cellulose for 5 days. Culture supernatants were harvested, dried and re-suspended before proteins were run on a SDS-PAGE gel and silver stained. Arrows indicate proteins secreted by the *pkaA* mutant but not by the wild-type strain.
