# Supplementary material for: Aspergillus nidulans protein kinase A plays an important role in cellulase production
Source: Biotechnol Biofuels. 2015 Dec 18;8:213. doi: 10.1186/s13068-015-0401-1 (PMC4683954; doi:10.1186/s13068-015-0401-1)
Supplement: Supplementary file 5 — 10.1186/s13068-015-0401-1 List of the primer pair used in this work. [file 13068_2015_401_MOESM4_ESM.docx]

**Additional file 4: Table S4.** List of the primer pair used in this work.

| Primer | Sequence |
| --- | --- |
| pRS426-5' PKA UTR F | TAACGCCAGGGTTTTCCCAGTCACGACGTTCTGAAGCCCGATACAACC |
| PKA Spacer GFP R | AAAGTTCTTCTCCTTTACTCATTCCCCGTGTTCCGAAATCGGGGAACAGGTGACCG |
| PyrG 3 UTR PKA F | AAGAGCATTGTTTGAGGCGAATTCACCCTCTAACGAGTGATG |
| PKA 3 UTR-pRS426 R | GCGGTTAACAATTTCTCTCTGGAAACAGCTCTAAGGCAGGCAGTTCTCG |
| Afu PyrG RV FGSC | GAGCAGCGTAGATGCCTCGACC |
| GFP Ve 3 Afu RV | CTCAGACAGAATACGCCAAGCTTG |
| pRS426-5' snfA UTR F | GTAACGCCAGGGTTTTCCCAGTCACGACGTGGAGATGGAAGTCGAAAGG |
| CreA GFP | ATAGACATGCCGTCACATGG |
| Afu pyrG pCRS60 R | GAGCAGCGTAGATGCCTCGACC |
